# Role of TRIM24 in the regulation of proteasome-autophagy crosstalk in bortezomib-resistant mantle cell lymphoma

**DOI:** 10.1038/s41420-025-02355-6

**Published:** 2025-03-17

**Authors:** Corentin Bouvier, Maria Gonzalez-Santamarta, Núria Profitós-Pelejà, Marc Armengol, Grégoire Quinet, Quentin Alasseur, Laurie Ceccato, Wendy Xolalpa, Raimundo Freire, Julie Guillermet-Guibert, Karine Reybier, Anne-Marie Caminade, Hans C. Beck, Ana Sofia Carvalho, Rune Matthiesen, Jean Christophe Rain, James D. Sutherland, Rosa Barrio, Gaël Roué, Manuel S. Rodriguez

**Affiliations:** 1 Laboratoire de Chimie de Coordination (LCC) CNRS-UPR8241, Toulouse, 31077 France; 2 Lymphoma Translational Group, UBIRed, Josep Carreras Leukaemia Research Institute, 08916 Badalona, Spain; 3 Unidad de Investigación, Hospital Universitario de Canarias, Instituto de Investigación Sanitaria de Canarias (IISC), La Laguna, La Laguna, Santa Cruz de Tenerife Spain; 4 Instituto de Tecnologías Biomédicas, Universidad de La Laguna, La Laguna, 38200 Santa Cruz de Tenerife Spain; 5 BMolecular, Centre Pierre Potier, Toulouse, 31100 France; 6 Departamento de Ingeniería Celular y Biocatálisis, Instituto de Biotecnología, UNAM, 62210 Cuernavaca, Morelos Mexico; 7 Universidad Fernando Pessoa Canarias, Las Palmas de Gran Canaria, Spain; 8 Centre de Recherche en Cancerologie de Toulouse (CRCT), Inserm, CNRS, Université de Toulouse, Toulouse, 31100 France; 9 PharmaDev, UMR 152, Université de Toulouse, IRD, UT3, 31400 Toulouse, France; 10 Department of Clinical Biochemistry, Odense University Hospital, Odense, Denmark; 11 Computational and Experimental Biology Group, iNOVA4Health, Nova Medical School, Facultade de Ciências Médicas, Universidade Nova de Lisboa, 1150-082 Lisboa, Portugal; 12 Hybrigenics Services, 1 rue Pierre Fontaine, 91000 Evry Genopole, France; 13 Center for Cooperative Research in Biosciences (CIC bioGUNE), Basque Research and Technology Alliance (BRTA), Bizkaia Technology Park, Building 801A, 48160 Derio, Spain

**Keywords:** Ubiquitin ligases, Lymphoma

## Abstract

Resistance to bortezomib (BTZ) represents a major bottleneck to continue using this proteasome inhibitor in the treatment of mantle cell lymphoma (MCL). In this study, we investigated the mechanisms by which TRIM24 (tripartite motif-containing 24), a ubiquitin ligase enriched in the ubiquitome of BTZ-resistant MCL cells, modulates proteasome-autophagy crosstalk. The localization and stability of TRIM24 were differentially influenced by the inhibition of proteasome or autophagy in MCL cells with acquired BTZ resistance (ZBR). Moreover, genetic deletion of the TRIM24 gene in ZBR (ZBR^TRIM24 KO^) effectively impaired cell proliferation without impacting the degradation of the proteasome by proteaphagy that is typically observed in BTZ-resistant cells. Notably, pre-treatment of ZBR cells with a proteolysis-targeting chimera (PROTAC) targeting TRIM24 (dTRIM24) successfully restored BTZ susceptibility, underscoring the critical role of TRIM24 in mediating resistance to proteasome inhibition. Interestingly, the combined apoptogenic activity of dTRIM24 and BTZ was preserved in a second BTZ-resistant clone (JBR) that lacks functional p53, indicating that this tumor suppressor is not required for the observed effect. Furthermore, we demonstrated that reducing TRIM24 protein levels in BTZ-resistant cells via dTRIM24 treatment restored proteasome activity, facilitating efficient apoptosis induction in cells exposed to the dTRIM24/BTZ combination. Mechanistically, dTRIM24 treatment promoted the formation of K48-linked ubiquitin chains and their association with proteasome subunits, specifically in BTZ-resistant cells. Taken together, these findings reveal that TRIM24 plays a pivotal regulatory role in the crosstalk between the proteasome and autophagy in BTZ-resistant MCL cells by modulating ubiquitin chain abundance, thereby influencing the activation of one or the other proteolytic pathway.

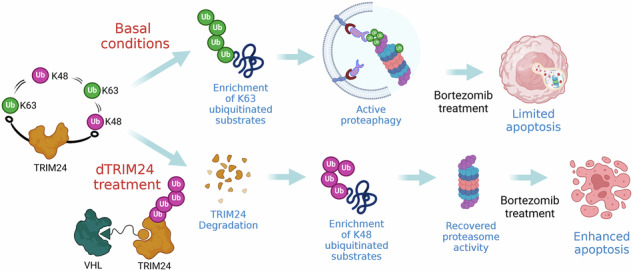

## Introduction

Mantle cell lymphoma (MCL) is a heterogeneous disease characterized by high genetic instability, variable progression, diverse behavior, and a wide range of treatment options, all of which pose significant challenges in achieving favorable long-term outcomes for patients [1, 2]. MCL is an aggressive form of non-Hodgkin lymphoma that exhibits a notable rate of relapse following treatment with the proteasome inhibitor (PI) bortezomib (BTZ), which is used not only in the clinical management of MCL but also for other hematological conditions such as multiple myeloma (MM) [3, 4].

BTZ acts by targeting the proteasome, more specifically the β5 catalytic subunit of the 20S core particle (CP), inhibiting its ability to degrade intracellular targeted proteins [5]. Different molecular mechanisms have been proposed to explain BTZ resistance in MCL and MM, for instance mutations/alterations in the β5 subunit, crosstalk with other proteolytic pathways, engagement of endoplasmic reticulum, oxidative stress response, epigenetic, and microenvironmental factors, among others, leading the scientific community to consider BTZ acquired resistance as a multifactorial event [6–9]. For this reason, understanding key molecular mechanisms regulating is crucial to develop new therapies to overcome the lack of response and relapse in cancer patients.

Post-translational modifications (PTMs) contribute to maintaining the natural balance of proteins inside the cells. Members of the ubiquitin family including ubiquitin (Ub) and ubiquitin like-proteins (UbL), such as LC3/GABARAP proteins, are directly implicated in the regulation of protein degradation by the two major intracellular proteolytic pathways, the Ub proteasome (UPS) and the autophagy lysosome (ALS) systems. Under certain conditions, those modifications can also regulate localization and activity of the modified targets.

Different enzymes regulate protein ubiquitylation. Ub-activating enzymes (E1), Ub-conjugating enzymes (E2) and Ub-ligases (E3), act in a coordinated manner to form Ub monomers or polymers on the target protein. The reverse reaction is mediated by deubiquitinating enzymes (DUBs) that remove or remodel already formed chains and confer high reversibility to this system [10]. Protein ubiquitylation most commonly occurs on a Lysine (K) residue, and as Ub itself contains 7 lysine residues, different Ub chain linkages can be formed. The most predominant form of Ub chains formed on substrates are K48 and K63 [11]. K48 Ub chains are known to drive proteasomal degradation while K63 are usually linked to regulation of signal transduction, DNA repair or autophagy degradation [10, 12–14].

The two major intracellular proteolytic pathways UPS and ALS are driven by two distinct systems of proteases. The 26S proteasomes are barrel-shaped complexes composed of a 20S core particle (CP) carrying three proteolytic activities in distinct subunits [β1 (caspase-like), β2 (trypsin-like) and β5 (chymotrypsin-like)] and one or two 19S regulatory particles (RP) capping both ends of the CP, in charge of recruiting ubiquitylated proteins [15]. By contrast, autophagy is initiated by the formation of the phagophore that engulfs cytoplasmic complexes or organelles and forms the mature autophagosome [10, 16]. This process is regulated by LC3/GABARAP proteins which are integrated into lipid membranes through conjugation to phosphatidyl ethanolamine (PE) [17]. Ubiquitylated targets are recognized by Ub binding domains present in autophagy receptors that also carry an LC3 interacting motif (LIR), facilitating their incorporation into autophagosomes [10, 16]. Targeted cargos are later degraded by hydrolases after the autophagosome-lysosome fusion. Several cargo receptors have been reported, and specific or redundant functions have been found for some of them. The prototype autophagy receptor p62/Sequestosome-1 (p62/SQSTM1) regulates multiple selective autophagy events and it has been implicated in various diseases [18, 19].

Although the UPS and the ALS are usually considered as independent processes, increasing evidence points to their interconnection [20–23]. An interesting compensatory mechanism is the proteasome inactivation by autophagy-mediated degradation, a process known as proteaphagy, described in multiple organisms, including mammals [16, 24, 25]. Using MCL cells with innate and acquired resistance to BTZ, we recently demonstrated that proteaphagy, mediated by the autophagy receptor p62/SQSTM1, is constitutively activated and contributes to BTZ resistance [24]. A concomitant enrichment of K63 Ub chains and Ub enzymes was also observed in BTZ-resistant cells. Among the top 5 Ub E3 enzymes found enriched in BTZ-resistant cells, TRIM24 (tripartite motif 24) belongs to a family of E3 ligases that have been involved in multiple cellular processes such as intracellular signaling, apoptosis, protein quality control, autophagy, carcinogenesis, being their dysregulation associated to the development of multiple diseases including cancer [26–28]. A well-known substrate of TRIM24 is the p53 tumor suppressor, which undergoes UPS-mediated degradation due to an evolutionary conserved interaction. Accordingly, TRIM24 targeting has been proposed to restore p53 activity [29]. In parallel, other TRIM proteins have been identified to be positive or negative modulators of p53 stability, making the TRIM/p53 axis an attractive therapeutic target in chemoresistant tumors [28].

Here, we explored the role of TRIM24 in the regulation of the UPS-ALS crosstalk and in the survival of MCL cells with acquired resistance to BTZ. We analysed TRIM24 stability, localization, its role in autophagy and cell viability in BTZ-resistant ZBR cells. ZBR^TRIM24 KO^ cells were used to demonstrate that TRIM24 is required for the optimal proliferation of MCL cells exposed to BTZ. The pharmacological reduction of TRIM24 using the PROTAC dTRIM24 [26] combined with BTZ induced a cooperative apoptosis in both p53-wild type and p53-mutated BTZ-resistant cells, and led to shift in ratio of K48/K63-linked Ub chains. Thus, our data support an important role for TRIM24 levels in the control of UPS/ALS crosstalk.

## Results

### Modulation of TRIM24 stability and localization in BTZ-resistant cells

**Fig. 1:**
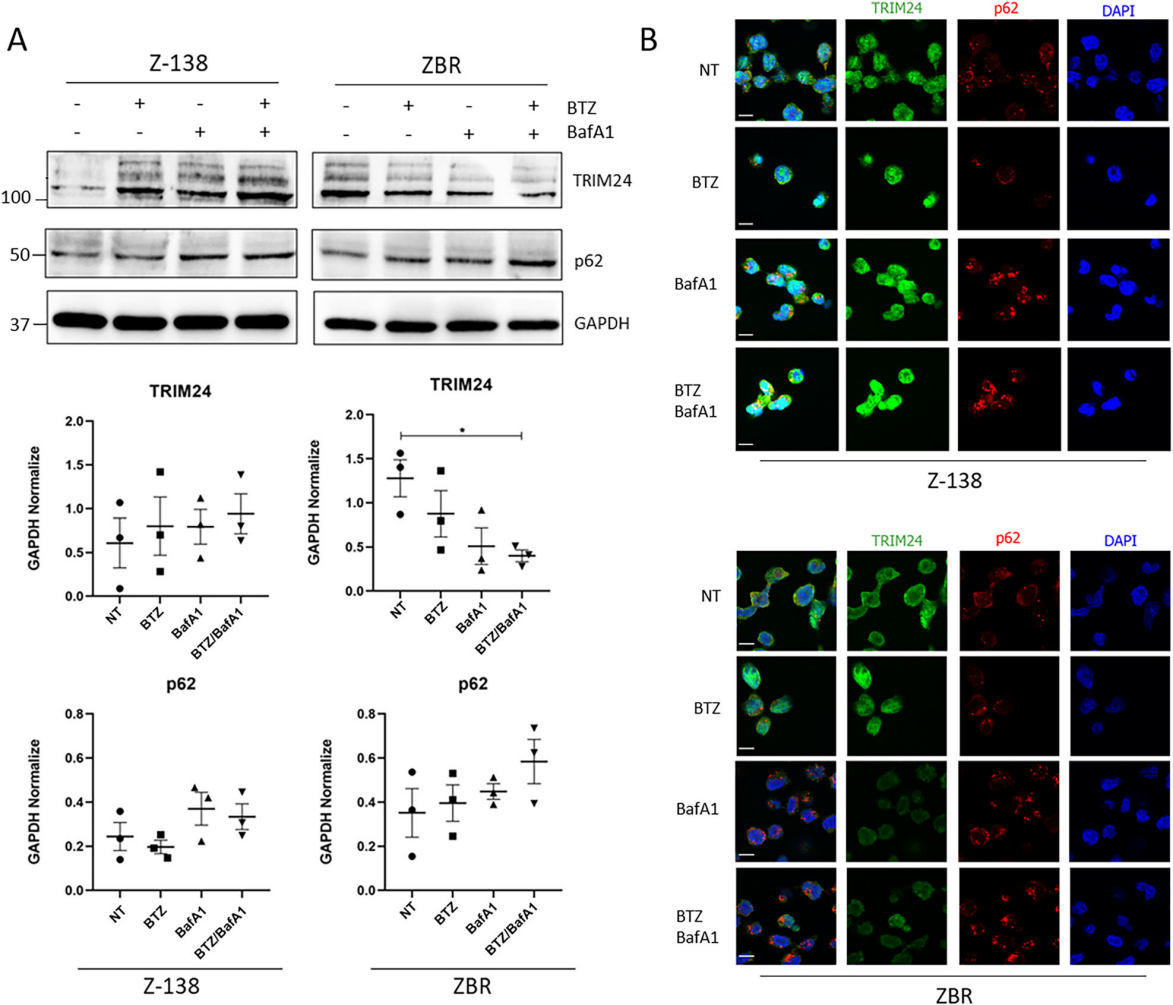
TRIM24 stability is differentially regulated in BTZ-resistant ZBR cells. **A** Z-138 and ZBR cell lines were treated with BTZ 10 nM, BafA1 20 nM, and the combined treatment for 8 h. WB analyses were carried out to detect the levels of TRIM24 and p62 proteins. Quantifications were performed using ImageJ software (*n* ≥ 3) and normalised against GAPDH values. Two-Way ANOVA tests with multiple comparisons were applied between conditions using the GraphPad software for statistical analysis and visualization. The data are presented as the means ± SEM. The ∗*p* < 0.05, ∗∗*p* < 0.01 or ∗∗∗*p* < 0.001 values were considered statistically significant. **B** Immunofluorescence images of Z-138 and ZBR cell lines treated with BTZ 10 nM, BafA1 20 nM and the combination for 8 h, using TRIM24 (green) and p62 (red) antibodies (Scale bar: 10 µm).

Using a Ub-trap-mass spectrometry (MS) approach, we previously showed that a permanently activated proteaphagy was associated with resistance to the proteasome inhibitor BTZ in MCL [24]. In the same data set we observed that TRIM24 was enriched in the ubiquitome of the BTZ-resistant MCL cell line, ZBR, concomitantly with K63 Ub chains, compared to the parental BTZ-sensitive Z-138 cells (Supplementary Fig. 1A–E). Since the link between these molecular features remains to be established, we investigated if the stability and localization of TRIM24 was distinct between the two cell lines. To analyse the stability of TRIM24, a pharmacological approach was chosen, using BTZ alone, or combined with the autophagy inhibitor BafA1. Our results showed that basal expression of TRIM24 was higher in ZBR than in Z-138 (Fig. 1A), in agreement with our previous Ub traps-MS study [24]. In Z-138 cells, treatment with BTZ and/or BafA1, stabilized TRIM24, as assessed by Western blot and indirect immunofluorescence detection (Fig. 1B). In sharp contrast, in ZBR cells a significant decrease in TRIM24 levels was observed when both proteasomal and autophagic processes were impaired (Fig. 1A, B). Interestingly, in an unrelated cell line (HeLa), the changes in TRIM24 followed the same patterns as in Z-138 under similar treatment conditions, suggesting that TRIM24 downregulation after inhibition of these proteolytic pathways was specific to BTZ-resistant MCL cells (Supplementary Fig. 2). Interestingly, the inhibition of both proteolytic pathways led to an increased accumulation of the proteaphagy receptor p62/SQTSM1 in ZBR when compared to Z-138, without changing the TRIM24/p62 co-localization in any of the studied cell lines (Fig. 1B and Supplementary Fig. 2). Immunofluorescence and nuclear-cytoplasmic fractionation followed by Western blot analysis further supported a distinct TRIM24 localization between the two cell lines, as shown by a broad nuclear staining in Z-138 cells, opposed to the cytoplasmic accumulation characterizing the ZBR cells (Supplementary Fig. 3). Altogether, these results indicate that TRIM24 localization and stability varied between BTZ-sensitive and BTZ-resistant MCL cells, suggesting that it could play distinct roles depending on the sensitivity to BTZ of the studied cells.

### TRIM24 is required for cell proliferation of MCL cells resistant to BTZ

**Fig. 2:**
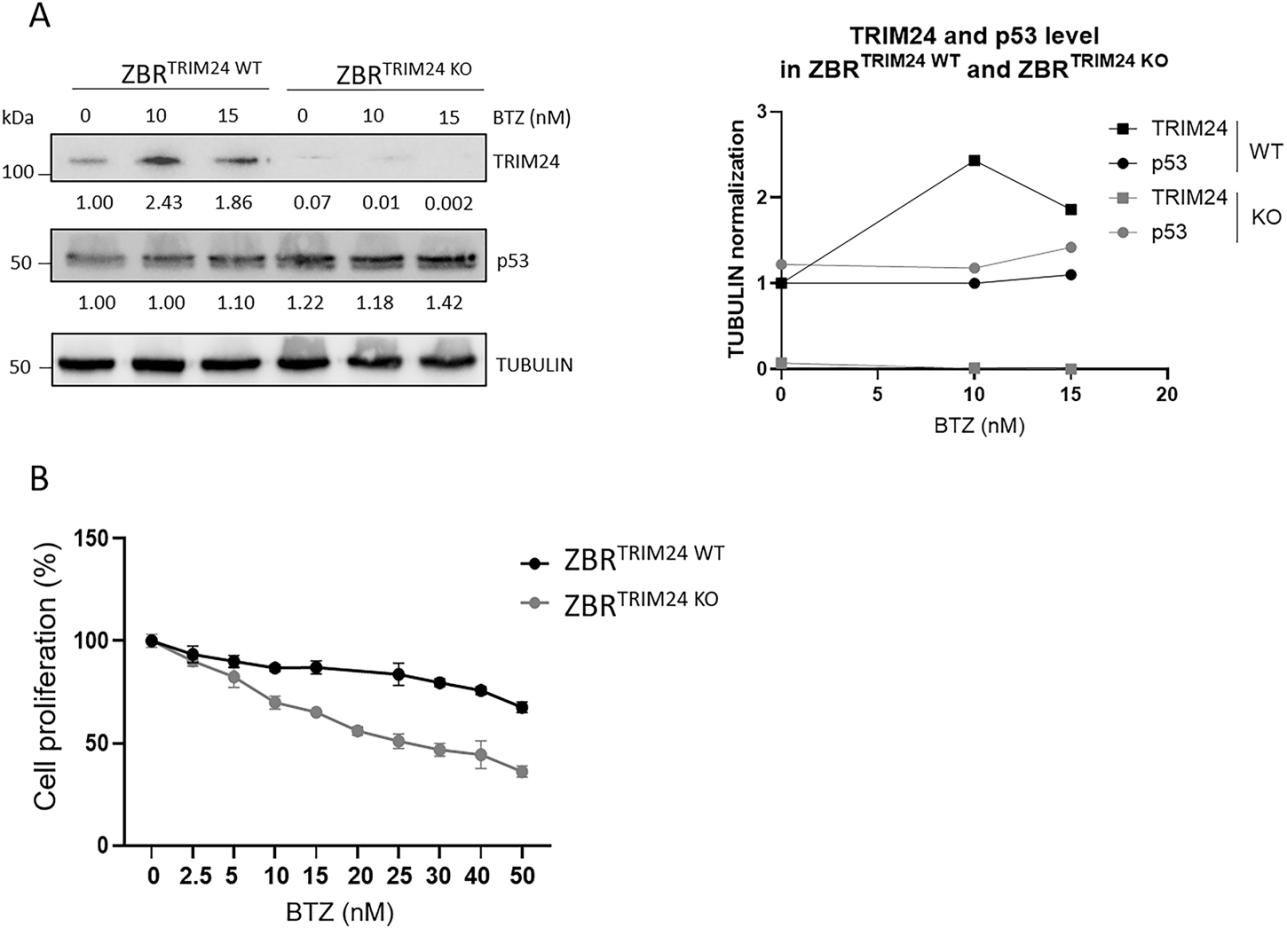
TRIM24 is required to regulate ZBR cell proliferation in response to BTZ. ZBR^TRIM24 WT^ and ZBR^TRIM24 KO^ cells were treated with the indicated doses of BTZ for 24 h. **A** WB using specific antibodies to detect TRIM24 and p53 from ZBR^TRIM24 WT^ or ZBR^TRIM24 KO^ cell extracts. Tubulin was used as loading control. **B** Cell proliferation assay was performed using MTT [3-(4,5-dimethylthiazolyl-2)-2,5-diphenyltetrazolium bromide] assay. The concentration required to reduce proliferation by 50% (IC_50_) was 71.64 nM for ZBR^TRIM24 WT^ and 24.44 nM for ZBR^TRIM2 4KO^. IC_50_ was calculated with the GraphPad Prism (GraphPad Software).

To characterize the role of TRIM24 in the regulation of cell survival of BTZ-resistant cells, a ZBR^TRIM24 KO^ cell line was engineered using CRISPR/Cas9 gene editing. In a ZBR subclone with a genetic deletion of *TRIM24*, increasing concentrations of BTZ failed to accumulate TRIM24 compared to parental cells (Fig. 2A). However, the tumor suppressor p53 was still accumulated in these cells upon BTZ treatment, underlining the presence of functional proteasomes despite the absence of TRIM24 (Fig. 2A). Cell proliferation in the presence of increasing concentrations of BTZ was drastically affected in ZBR^TRIM24 KO^ cells when compared to the ZBR^TRIM24 WT^ counterpart (Fig. 2B). The estimated BTZ IC_50_ values were 71.64 nM for ZBR^TRIM24 WT^ and 24.44 nM for ZBR^TRIM24 KO^, suggesting an almost complete recovery of BTZ sensitivity in ZBR^TRIM24 KO^ cells (Fig. 2B) [30]. Therefore, the genetic ablation of *TRIM24* appears to increase the sensitivity of ZBR cells to BTZ, suggesting the participation of this Ub ligase in adaptive drug resistance.

### Proteolysis of TRIM24 regulates apoptosis in both BTZ-sensitive and BTZ-resistant cells

**Fig. 3:**
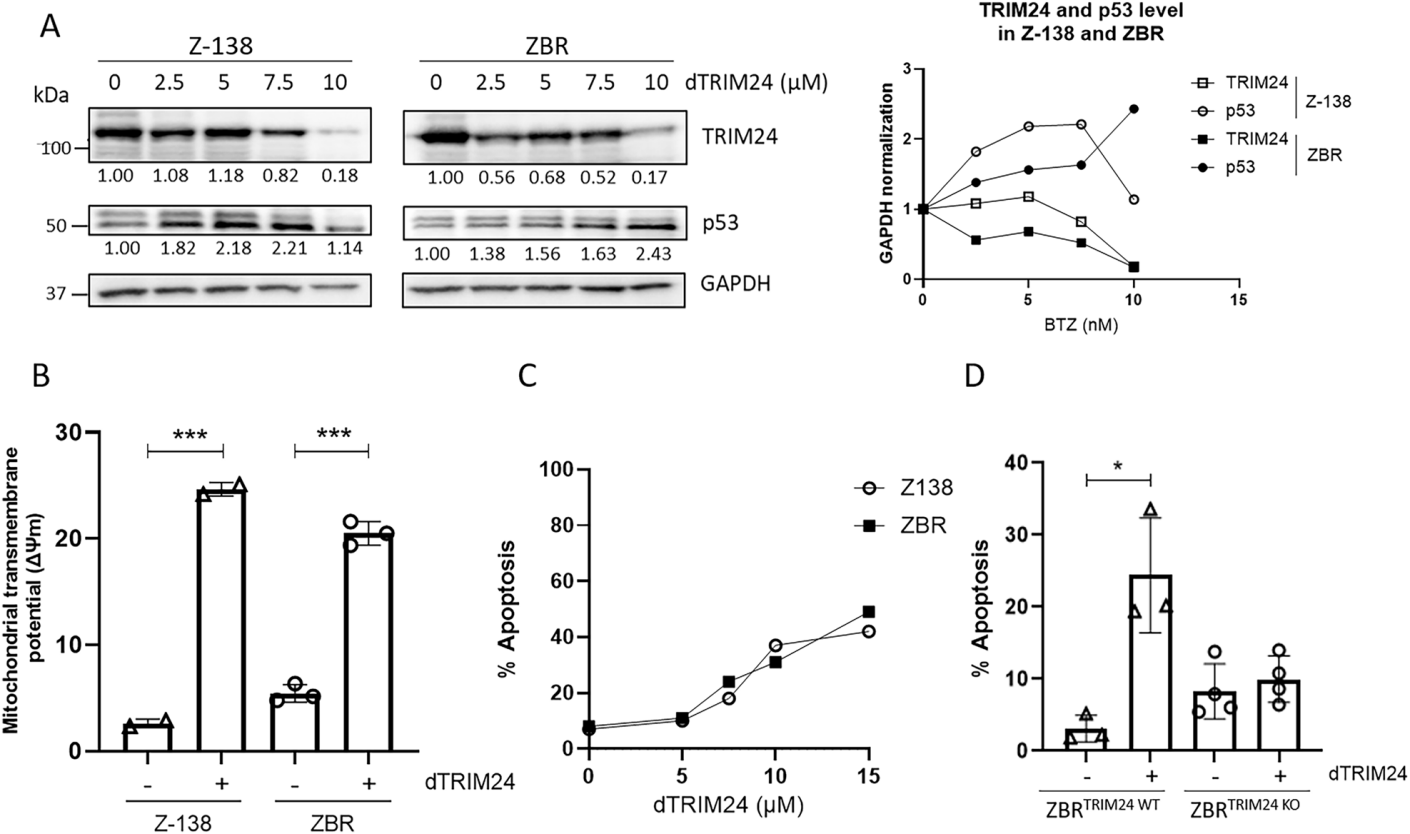
Degradation of TRIM24 triggers cell death in both Z-138 and ZBR cell lines. **A** *Right panel:* BTZ-sensitive Z-138, and BTZ-resistant ZBR cell lines were treated with 2.5, 5 and 10 µM dTRIM24 for 24 h. Levels of TRIM24 and p53 were analysed by Western blot. *Left panel:* Quantification of TRIM24 and p53 using GAPDH to normalize values. **B** Mitochondrial transmembrane potential after treatment of Z-138 and ZBR cells with 10 µM dTRIM24. *t*-test student were applied to compare two conditions, untreated and treated, using the GraphPad software for statistical analysis and visualization. The data are presented as the means ± SD. The ∗*p* < 0.05, ∗∗*p* < 0.01 or ∗∗∗*p* < 0.001 values were considered statistically significant. **C** Dose-dependent activity of dTRIM24 was determined in Z-138 and ZBR cells by cytofluorimetric quantification of apoptosis after a 24 h treatment, and IC_50_ values were calculated using the GraphPad software. **D** dTRIM24 induced apoptotic activity was measured in ZBR^TRIM24 WT^ and ZBR^TRIM24 KO^ cells. Significant (*) apoptosis was observed in ZBR^TRIM24 WT^ cells only.

To characterize the mechanisms by which TRIM24 regulates the survival of BTZ-resistant cells, a previously validated PROTAC targeting TRIM24 (dTRIM24) was applied to BTZ-sensitive and BTZ-resistant cell cultures, following previously reported conditions [26]. A dose-dependent evaluation of dTRIM24 in Z-138 and ZBR cells identified 10 µM as the optimal concentration of the PROTAC able to consistently reduce the levels of the Ub ligase in these MCL cells (Fig. 3A). As expected, cell treatment with dTRIM24 was accompanied by a dose-dependent accumulation of the TRIM24 target, p53, in both Z-138 and ZBR cells (Fig. 3A). Reduced p53 and GAPDH protein levels were observed in Z-138 at 10 µM of dTRIM24, suggesting the activation of apoptosis under those conditions. To analyse the impact of TRIM24 reduction on apoptosis onset in ZBR and Z-138 cells, we used flow cytometry to quantify the mitochondrial transmembrane potential loss upon exposure to dTRIM24 (Fig. 3B, C and Supplementary Fig. 4). Programmed cell death was efficiently detected in the presence of the PROTAC in both Z-138 and ZBR cell lines, as reflected by a 20-25% increase in mitochondrial transmembrane potential loss and reaching >40% apoptosis at the maximal dose tested (15 µM), with no visible difference between the parental cell line and its derivative (Fig. 3B, C and Supplementary Fig. 4). To evaluate the specificity of the cell killing effect of dTRIM24, we compared the activity of the PROTAC in ZBR^TRIM24 KO^ cells to their parental counterparts (Fig. 3D and Supplementary Fig. 4). While basal levels of apoptosis were consistently higher in ZBR^TRIM24 KO^ cells, no response to dTRIM24 was detected, contrasting with the significant proportion of parental ZBR cells undergoing cell death, and indicating that dTRIM24 effect was specific at the tested dose (10 µM) (Fig. 3D and Supplementary Fig. 4).

**Fig. 4:**
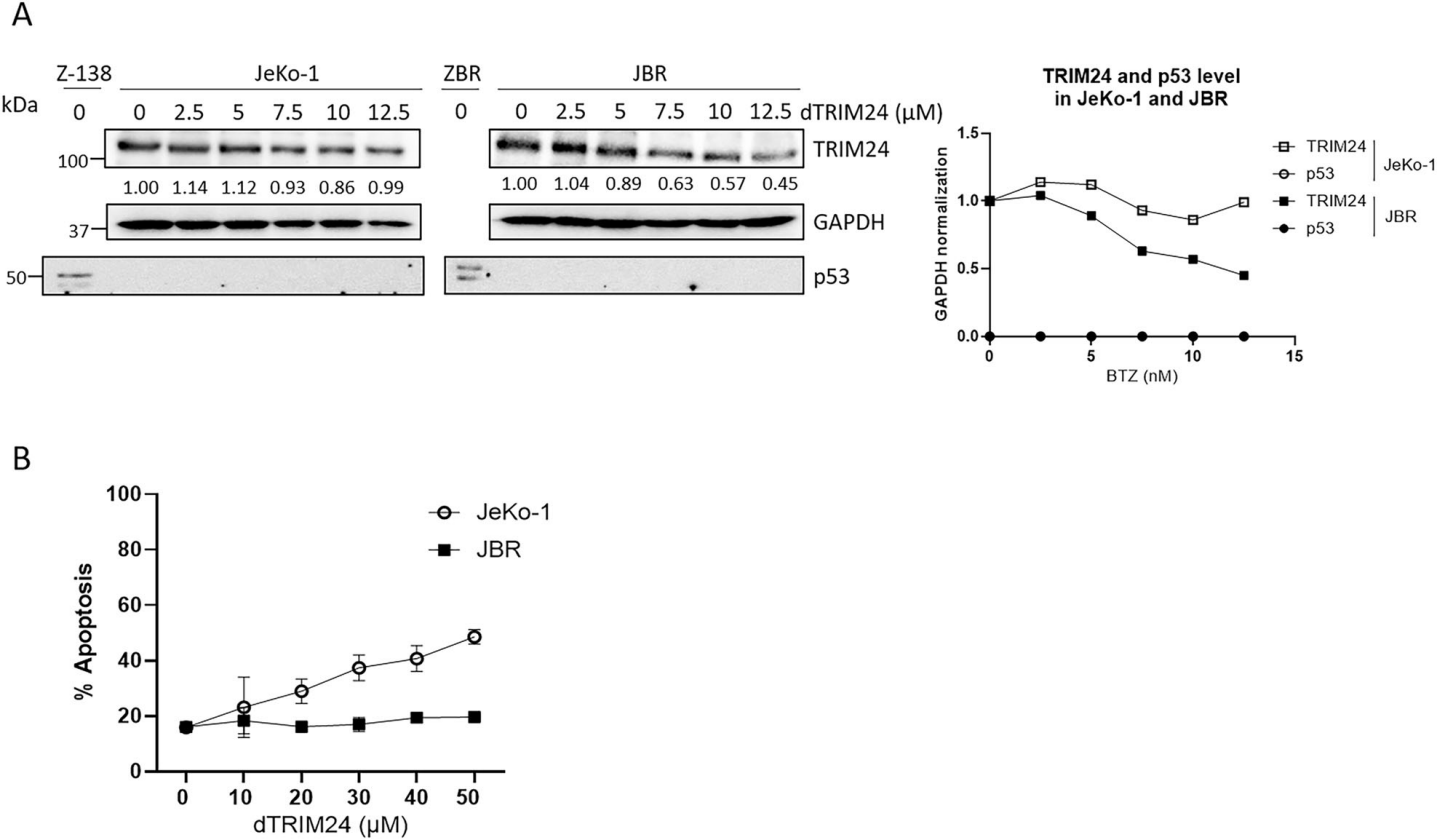
dTRIM24 requires a fully functional proteasome but not p53 to efficiently drive apoptosis. **A** BTZ-sensitive JeKo-1, and BTZ-resistant JBR cell lines were treated with 2.5, 5, 7.5, 10 and 12.5 µM dTRIM24 for 24 h. *Left panel:* WB analyses were carried out to detect the protein levels of TRIM24 and p53. *Right panel:* Quantification of TRIM24 using GAPDH to normalize values. **B** Dose-dependent activity of dTRIM24 was determined in Jeko-1 and JBR cell lines by cytofluorimetric quantification of apoptosis after a 24 h treatment, and IC_50_ values were calculated using the GraphPad software.

Since both genetic ablation of TRIM24 and dTRIM24 treatment increased the levels of p53, we explored the relevance of this tumor suppressor in the triggering of apoptosis. To answer this question we took advantage of a second BTZ-resistant clone, JBR, and its parental counterpart JeKo-1, both lacking a functional p53 locus due to 17p deletion [30]. When used at doses ranging from 7.5–12.5 µM, dTRIM24 could reduce TRIM24 levels which was concomitant with the apoptotic affect observed after 24 h of treatment in JeKo-1 and JBR cells (Fig. 4A, B and Supplementary Fig. 5). However, using the same concentration or even increasing to 50 µM, dTRIM24 treatment was unable to trigger JBR cell death, while it could induce up to 50% apoptosis in parental JeKo-1 cells (Fig. 4B and Supplementary Fig. 5). Thus, these results indicate that BTZ-resistance somehow regulates dTRIM24-induced apoptosis in MCL cells lacking p53.

### dTRIM24 treatment sensitizes ZBR and JBR cells to bortezomib in vitro and in vivo

**Fig. 5:**
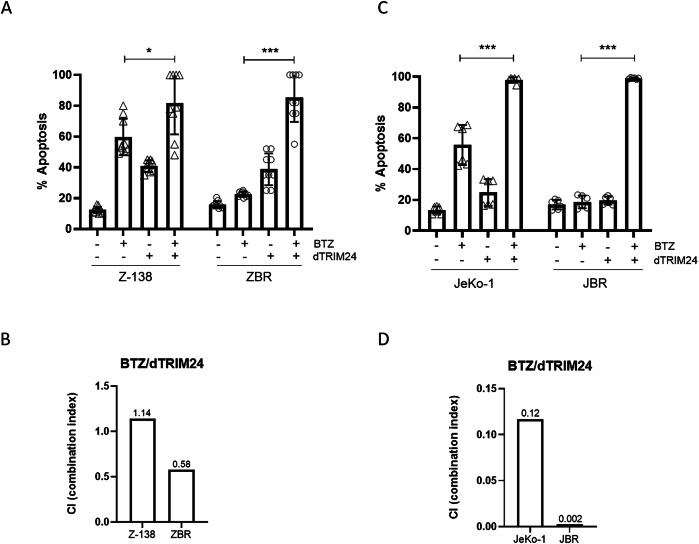
Reduction of TRIM24 levels recover BTZ sensitivity in resistant cells. **A** Cytofluorimetric (FACS) determination of the % of apoptotic cells after a 24 h treatment with 5 nM BTZ, 10 µM dTRIM24 and the combination of both drugs, in Z-138 and ZBR cell lines. *t*-test student were applied to compare combined treatment using the GraphPad software for statistical analysis and visualization (*n* ≥ 9). The data are presented as the means ± SD. The *p* values > 0.05 were considered statistically non-significant (ns). **B** Combination index (CI) of BTZ/dTRIM24 treatment in Z-138 and ZBR cells, calculated according to materials and methods. CI < 0.8 is indicative of a synergistic effect in the BTZ-resistant (ZBR) cell line. **C** FACS determination of the % of apoptotic cells after 24 h treatment with 15 nM BTZ, 20 µM dTRIM24 and the combination of both drugs, in JeKo-1 and JBR cell lines. *t*-test student were applied to compare combined and BTZ treatment using the GraphPad software for statistical analysis and visualization (*n* ≥ 6). The data are presented as the means ± SD. The *p < 0.05, **p < 0.01 or ***p < 0.001 values were considered statistically significative. **D** CI values corresponding to BTZ/dTRIM24 treatment in JeKo-1 and JBR cells, calculated according to materials and methods. CI < 0.12. and 0.002 are indicative of a synergistic effect in both cell lines.

To explore if the proteolysis of TRIM24 could resensitize ZBR cells to apoptosis induced by BTZ, we evaluated the effect of the dTRIM24/BTZ combination therapy considering different concentrations of both agents and using Z-138 cells for comparison. After 24 h, while the individual treatments (dTRIM24 10 µM and BTZ 5 nM) induced notable levels of apoptosis in Z-138, we observed that dTRIM24 alone was able to maintain its activity at comparable levels in both ZBR and Z-138 cells (Fig. 5A and Supplementary Fig. 6). Interestingly, the dTRIM24/BTZ combination achieved to trigger up to 80% apoptosis in ZBR cells, showing a significant improvement of activity when compared to treatments with each compound alone. In contrast, in Z-138 cells this combinatorial treatment only modestly increased apoptosis when compared to individual compounds (Fig. 5A and Supplementary Fig. 6). Accordingly, the combination index of the two drugs when used at 5 nM (BTZ) and 10 µM (dTRIM24) was 0.58 in ZBR cells, indicating a synergistic action, but was > 1.0 in Z-138 cells, indicating additive effect in bortezomib-sensitive cells (Fig. 5B). To evaluate if the dTRIM24/BTZ treatment was also efficient in JBR cells compared to JeKo-1, these cell lines were exposed to 15 nM BTZ and 20 µM dTRIM24. Individual treatments induced apoptosis in JeKo-1, but failed to do so in JBR cells. Conversely, the dTRIM24/BTZ combination enhanced apoptosis in both JeKo-1 and JBR, with this effect being significantly superior in the latter (Fig. 5C and Supplementary Fig. 6). Supporting this observation, the combination index of the two drugs was 0.12 in JeKo-1 cells and 0.002 in JBR cells, indicative of a strong synergistic effect of the two drugs in both cell lines and mostly in BTZ-resistant JBR (Fig. 5D).

**Fig. 6:**
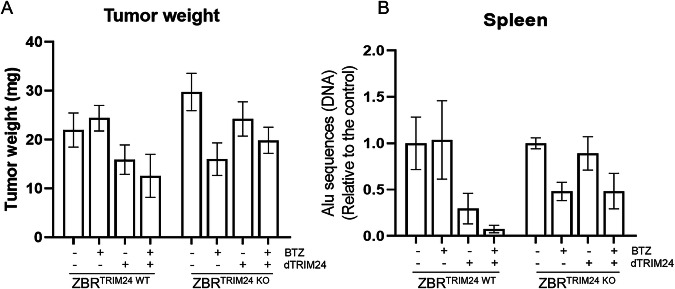
dTRIM24 treatment improves BTZ sensitivity in vivo. Two immunocompetent xenograft models of MCL derived from ZBR^TRIM24 WT^ and ZBR^TRIM24 KO^ cell lines were generated using the chick embryo chorioallantoic membrane (CAM) method. **A** Tumor-bearing CAMs were treated twice weekly with BTZ +/- dTRIM24 and at ED16 tumors were weighed. **B** ZBR metastatic potential was measured by quantification of human Alu sequences in the spleen of *n* = 3 representative chick embryos xenografted with TRIM24^WT^ and TRIM24^KO^ and subjected to the different treatments. Values are referred to vehicle-treated samples.

To further support the activity and specificity of the dTRIM24/BTZ combination in an in vivo setting, we developed two immunocompetent xenograft models of MCL derived from ZBR^TRIM24 WT^ and ZBR^TRIM24 KO^ cell lines using the chick embryo chorioallantoic membrane (CAM) method (Fig. 6) [31]. After engraftment of MCL cells, the tumors were exposed twice to BTZ +/- dTRIM24 or an equivalent volume of vehicle. On day 16 post-egg fertilization, the chick embryos were sacrificed, and tumors were weighed. As shown in Fig. 6A, the exposure of TRIM24-depleted tumors, but not their wild type counterparts, to BTZ, led to a 43% decrease in tumor weight, while this effect was not improved by the addition of dTRIM24. Conversely, the combination of both agents achieved a 48% tumor growth inhibition in TRIM24^WT^ tumors, superior to the 23% achieved by dTRIM24 monotherapy, and in agreement with our in vitro results. Of importance, a marked reduction in ZBR metastatic potential was observed in both the TRIM24^WT^ tumors treated with the combination and in the TRIM24^KO^ xenografts exposed to BTZ, as observed by a 90% and 55% drop (respectively) in the abundance of human Alu sequences in the spleen of the chick embryos (Fig. 6B). These results confirm a crucial role of TRIM24 in the response of MCL propagation to BTZ therapy.

### dTRIM24 enhances proteasome activity

**Fig. 7:**
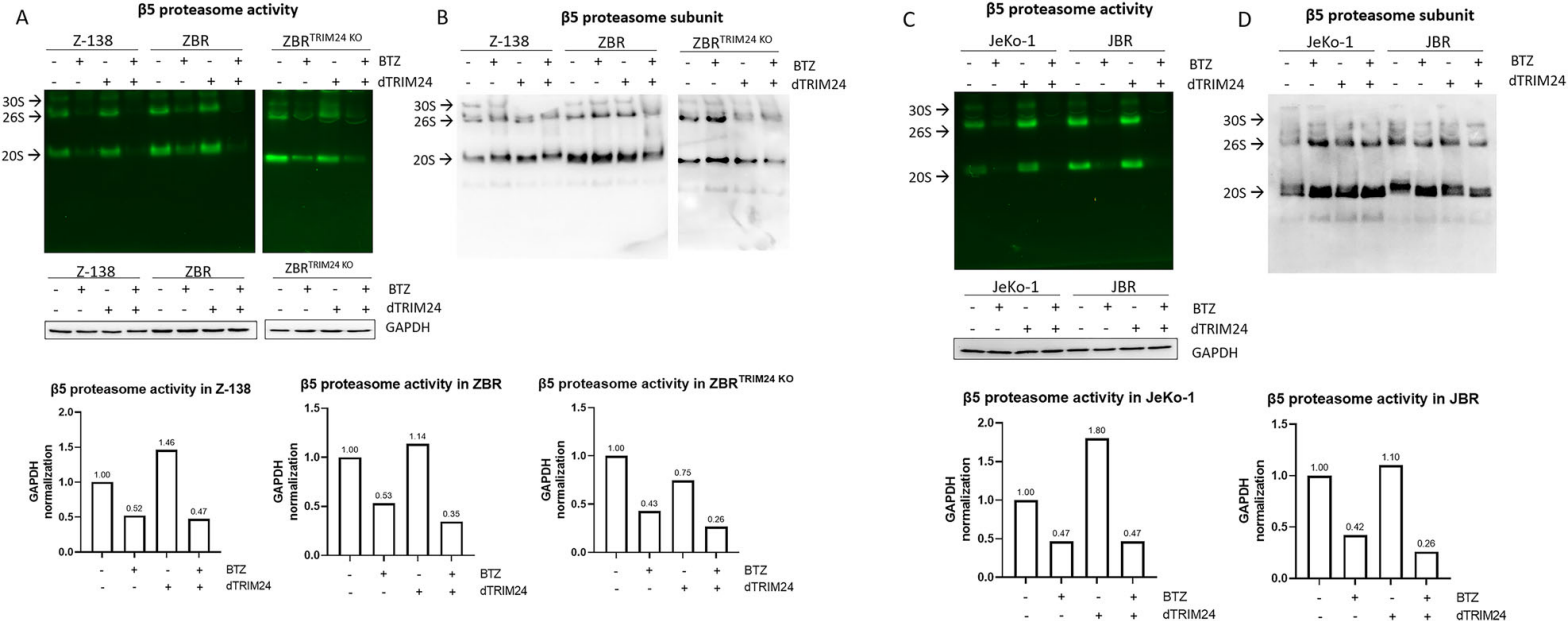
dTRIM24 treatment enhances proteasome activity. **A** In-gel proteasome activity assays were performed with extracts of Z-138, ZBR and ZBR^TRIM24 KO^ cells treated or not with dTRIM24. The specificity of the fluorescent reporter peptide was assessed using cell extracts treated BTZ. Proteasomal activity was normalised using GAPDH detected by Western blot. **B** The same gel used in “A” was western-blotted to detect the β5 proteasome subunit. 20S, 26S and 30S complexes, as indicated. **C** In-gel proteasome activity assays were performed with extracts of JeKo-1 and JBR cells treated or not with dTRIM24. The specificity of the fluorescent reporter peptide was assessed using cell extracts treated with BTZ. Proteasomal activity was normalised using GAPDH detected by Western blot. **D** The same gel used in “A” was western-blotted to detect the β5 proteasome subunit. 20S, 26S and 30S complexes, as indicated.

In order to investigate if the sensitivity to BTZ in ZBR cells was recovered after dTRIM24 treatment, the activity of the proteasome was measured in native gels using extracts from cells treated with dTRIM24, BTZ or both (Fig. 7A and Supplementary Fig. 7A). The intensity of the fluorescence of the peptide substrate revealed that dTRIM24 treatment increased the activity of the 20S and 26S proteasome complexes, with quantification based on normalization of fluorescence versus GAPDH levels determined by western blotting. The activity increase after dTRIM24 treatment was not associated with an increased β5 protein level (Fig. 7B and Supplementary Fig. 7B). As expected, the dTRIM24 treatment did not enhance proteasome activity in ZBR^TRIM24 KO^ cells. The observed proteasome activity was specific since it was inhibited with BTZ. However, a residual proteasome activity was observed after BTZ treatment in Z-138, ZBR and ZBR^TRIM24 KO^ cell lines. Since the dTRIM24/ZBR combination resulted in a more effective induction of apoptosis in JeKo-1 and JBR cell lines, we quantified the proteasome activity in those cells upon the different treatments (Fig. 7C and Supplementary Fig. 7C). The 20S and 26S proteasome activity was systematically enhanced by the dTRIM24 treatment in JeKo-1 and JBR cells. Under the conditions tested, the β5 protein levels were not affected (Fig. 7D and Supplementary Fig. 7D). In JeKo-1 and JBR cells, the inhibition of the proteasome activity with BTZ was more efficient, with almost undetectable activity after dTRIM24/BTZ treatment, which is in agreement with the efficient apoptosis induced in these cell lines upon exposure to this combination.

### TRIM24 does not directly regulate proteaphagy

**Fig. 8:**
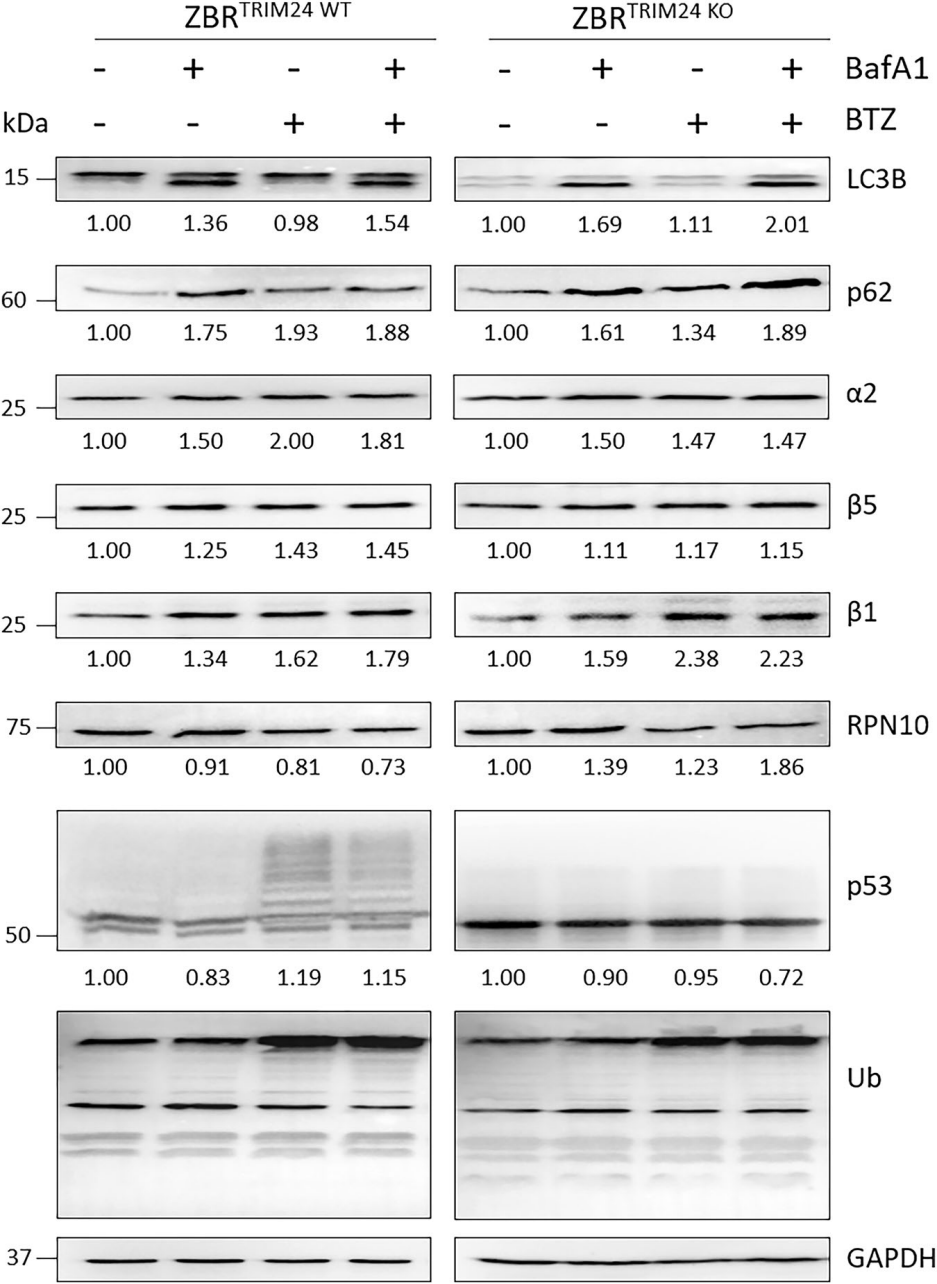
TRIM24 does not directly regulate proteaphagy in ZBR cells. ZBR and ZBR^TRIM24 KO^ cell lines were treated with 20 nM BafA1, 20 µM BTZ or the combined treatment for 6 h. Western blot analyses were carried out to detect LC3B, p62, a2, β5, β1 and RPN10 proteasomal subunits, total ubiquitination and p53. Quantifications were performed using ImageJ software (*n* ≥ 3) and normalised against GAPDH values. Ratios changes were calculated using untreated conditions as control (1fold).

Although the above evidence did not indicate that dTRIM24 had an impact in the recovery of the β5 protein level, we checked whether the total absence of TRIM24 could affect proteaphagy ignition in the presence or absence of BTZ (Fig. 8 and Supplementary Fig. 8). To achieve this, we compared the levels of proteaphagy in ZBR and ZBR^TRIM24 KO^ cell lines, upon treatment with BafA1, BTZ or both agents. Cell extracts were analysed by Western blot for distinct autophagy factors and proteasome subunits. As shown on Fig. 8, the levels of LC3B were reduced in the ZBR^TRIM24 KO^ compared to the parental ZBR. Nevertheless, the lipidated form of LC3B was detected in both cell lines after BafA1 treatment. The proteaphagy receptor p62 and proteasome subunits α2, β5, β1 and RPN10 were equally accumulated, indicating that proteaphagy was comparable in both cell lines. Total ubiquitination levels were also similar in both cell lines under the conditions tested. In contrast, ubiquitinated p53 was only observed in the parental ZBR cell when BTZ was used, confirming the importance of TRIM24 in the regulation of the stability of this tumor suppressor. Thus, these results suggested that TRIM24 regulates basal levels of autophagy, but with little or no effect on proteaphagy.

### dTRIM24 enhances K48 and K63 ubiquitination, increasing the binding of p62, β2 and β5 proteasomal subunits to enriched Ub chains only in BTZ-resistant MCL

**Fig. 9:**
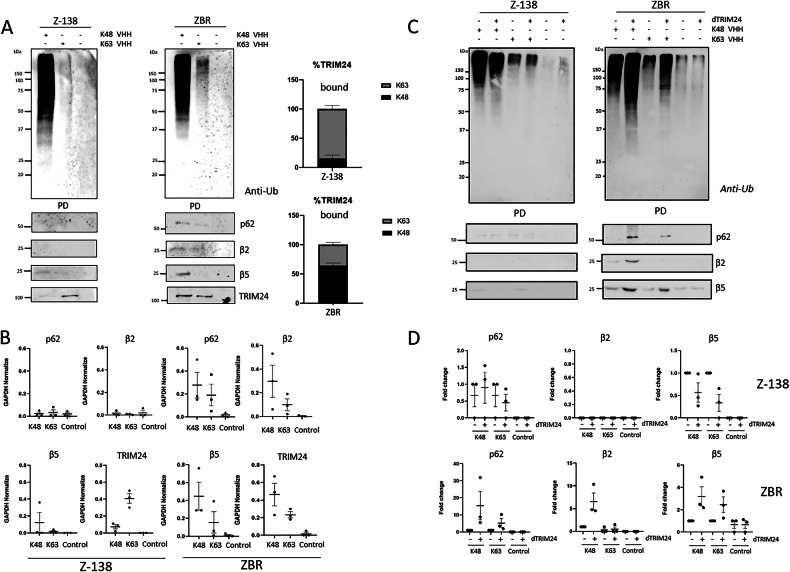
TRIM24 depletion increases K48 and K63 ubiquitination and their binding to p62, β2 and β5 proteasomal subunits in ZBR cells. **A** Specific nanobodies were used to capture K63 or K48 ubiquitin chains under basal conditions (no treatment). Anti-Ub is used to detect the ubiquitin from the pull-down (PD) fraction. WB analyses to detect TRIM24, p62, β2 and β5 proteasomal subunits levels in the PD fraction. **B** Quantifications were performed using ImageJ software (*n* ≥ 3) and normalised against GAPDH values. GraphPad software was used for statistical analysis and visualization. Graphics were made using GraphPad Prism Software. Control condition (without nanobody) was used. Proteins associated to K48 or K63 chain-specific nanobodies are displayed in the graphs (*n* = 3). **C** Z-138 and ZBR cell lines were with or without 10 µM of dTRIM24 treatment for 8 h. Precipitations were performed using K48 or K63 ubiquitin chain-specific nanobodies. Anti-Ub was used to detect by Western blot ubiquitylation pull-down (PD) fraction. WB were performed to detect TRIM24, LC3B, p62, β2 and β5 proteasomal subunits. **D** Quantifications were performed using ImageJ software (*n* ≥ 3) and normalised against GAPDH values. Graphics were made using GraphPad Prism Software. Control (without nanobody) values were efficiently low. Proteins associated to K48 or K63 chain-specific nanobodies are displayed in the graphs (*n* = 3).

**Fig. 10:**
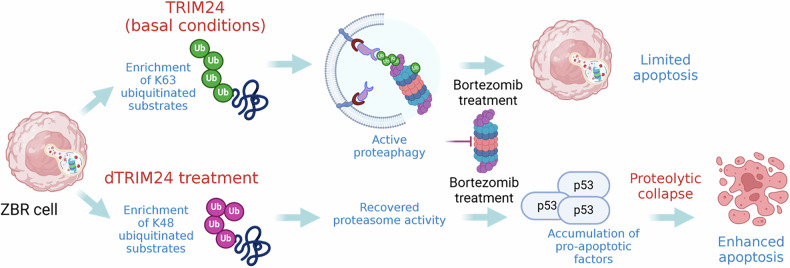
Schematic representation of the hypothetical mechanism of action of TRIM24 in ZBR cells. Under basal conditions TRIM24 contributes to activate proteaphagy by promoting K63 ubiquitin chain formation, contributing to the survival and low sensitivity to BTZ of ZBR cells. dTRIM24 treatment significantly increases K48 ubiquitin chain formation as compared to K63 ubiquitin chains in ZBR cells, reactivating the proteasome. The addition of BTZ to the dTRIM24 treatment results in the accumulation of proapoptotic factors such as p53 driving an efficient apoptosis. Figure created with BioRender.com.

In order to elucidate the possible mechanism of action by which dTRIM24 treatment efficiently restores the sensitivity to BTZ in resistant cells, we explored if TRIM24 could be implicated in the regulation of Ub chains. By using K48- or K63-Ub chain-specific nanobodies in pulldown assays, we could confirm our MS data indicating that there were more K63-Ub chains in ZBR than in Z-138 cells in normal conditions (Fig. 9A and Supplementary Fig. 1). Interestingly, while TRIM24 was mainly associated to K63-Ub chains in Z-138 cells, this Ub ligase was more associated to K48 chains in ZBR cells (Fig. 9A). In sharp contrast, under these conditions the autophagy receptor p62 and proteasome subunits β5 and β2 were better bound to both chain types in ZBR cells (Fig. 9A, B). The interaction between both K48- and K63-Ub chains and these proteins implicated in proteaphagy was almost undetectable in Z-138 cells (Fig. 9A, B), suggesting that proteaphagy was not active in the BTZ-sensitive cells. Strikingly, the PROTAC-mediated degradation of TRIM24 resulted in a massive accumulation of Ub chains in ZBR but not in Z-138 cells (Supplementary Fig. 9B). In ZBR cells treated with dTRIM24, K48-Ub chains were highly enriched compared to K63-Ub (Fig. 9C, D). This enrichment favored in particular the interaction of K48-Ub chains with p62 and β5 and β2 proteasome subunits in ZBR cells only (Fig. 9C, D). Nevertheless, some binding of K63-Ub chains to p62 and β5 could be also observed in ZBR cells. Taken together, these results highlight the regulatory role of TRIM24 on controlling the abundance and ratios of K48- and K63-Ub chains, which influence the targeting of substrates to the proteasome or autophagy (Fig. 10).

## Discussion

Multiple evidences support a crosstalk between the UPS and ALS systems under distinct physiologic and pathologic conditions. This coordination presumes the existence of cellular factors that will work as “sensors” to connect both proteolytic systems under distinct situations [20–22]. Recently, it was demonstrated that in MCL cells refractory to BTZ therapy, a permanently activated proteaphagy contributes to inactivate proteasomes, explaining at least in part why this treatment loses efficacy, as evident from patient-derived cell lines [24]. Another interesting feature identified using the TUBEs-MS approach was the enrichment of TRIM24, whose aberrant expression has been already associated with different cancer types and has been proposed as a cancer prognostic factor, associating increased proliferation with oncogenic transformation and aggressive malignant phenotypes [32–36]. TRIM24 has also been found to mediate chemoresistance in gastric cancer and glioblastoma [36, 37]^,^. The exact mechanisms by which TRIM24 contributes to developing different pathologic conditions is not fully understood. TRIM24 appears to play several roles, acting as co-regulator of transcription [38] but also as a negative regulator of p53 due to its Ub E3 ligase activity [29]. Interestingly, the silencing of another Ub E3, TRIM28, has already been shown to enhance sensitivity to BTZ in B-cell non-Hodgkin lymphoma cells resistant to this agent [39]. TRIM28, a known transcriptional regulator in the DNA damage response, is also an Ub E3 Ub ligase that negatively regulates p53 by promoting its ubiquitylation [40].

In this work, we conclude that the Ub-ligase TRIM24 can contribute to switching from one proteolytic system to the other by regulating the abundance of K48- and K63-Ub chains. Different lines of evidence support this notion. First, the basal level of TRIM24 is higher in ZBR than in Z-138 cells. Second, BTZ, BafA1 or the combined use of these drugs, lead simultaneously to decrease TRIM24 levels and increased p62/SQTSM1 expression in ZBR cells. In this BTZ-resistant cell line, we show that protein levels of TRIM24 are proportionally inverse with the level of p62/SQTSM1, which conditions the activity of the proteaphagy pathway. A future challenge is to understand when p62 works as proteaphagy receptor, and when it is a substrate for the proteasome. Interestingly, in BTZ-sensitive cells Z-138 (or in HeLa cells) TRIM24 accumulates after pharmacological inhibition of the proteasome and autophagy. In addition, differences in TRIM24 localization are observed between Z-138 and ZBR cells, with the enhanced cytoplasmic distribution suggesting distinct functions for this Ub E3 ligase in the BTZ-resistant ZBR cells. Third, the reduction of TRIM24 levels using dTRIM24 changes the ratio of K48/K63 ubiquitin chains which enhances proteasome activity and favors the recovery of the sensitivity to BTZ in resistant cells. Supporting the crucial role of TRIM24 in the viability of ZBR cells, genetic knockout of this Ub ligase compromised cell proliferation in response to BTZ.

Altogether, our evidence supports the existence of a mechanism by which TRIM24 is regulating the abundance of K48- and K63-Ub chains found in ZBR cells. In basal conditions, K63-chains are more abundant in ZBR compared to Z-138 cells, and this could favor autophagy processes. On the contrary dTRIM24 enhances the presence of K48-Ub chains, which may drive more targets to the proteasome. Although K63-chains are also accumulated after the dTRIM24 treatment, these are less abundant than K48 chains that favor the activity of the proteasome. The dramatic accumulation of K48-Ub chains after the dTRIM24 treatment in ZBR cells suggest that TRIM24 could be a master regulator of other Ub ligases, such as other TRIM ligases and UBR enzymes implicated in the N-end rule pathway identified using the TUBE-MS approach [24]. We can also speculate that TRIM24 could negatively regulate the stability or activity of de-ubiquitinating enzymes (DUBs) that might result in the accumulation of certain Ub chain-types. In fact, the abundance of some DUBs was also altered in the TUBE-associated proteome of ZBR cells [24]. Investigating the role of these Ub enzymes in the regulation of proteaphagy, Ub chain formation and activity of the proteasome can help us to better understand the response/resistance to BTZ and the mechanisms of crosstalk between these major proteolytic systems.

The dTRIM24 treatment of ZBR cells or the absence of TRIM24 in the ZBR^TRIM24 KO^ cells favors the accumulation of p53 in these cell lines, suggesting the contribution of this tumor suppressor to the apoptotic response observed in both cell lines. However, p53 does not seem to be the only tumor suppressor playing a role in driving apoptosis after dTRIM24/BTZ treatment since in JeKo-1 and JBR cells lacking a functional p53, this process is very efficient. Reduced levels of LC3B suggest that basal autophagy is affected in the ZBR^TRIM24 KO^. However, proteaphagy is not significantly affected since proteasome subunits are accumulated equally well in ZBR^TRIM24 KO^ and the parental ZBR cells after autophagy inhibition [24].

Blocking proteaphagy by interfering with the autophagy receptor p62 seems like it would be an interesting approach to recover BTZ sensitivity [16, 24]. However, our data indicate that there are other ways to regulate BTZ response such as regulating the abundance of TRIM24 with dTRIM24, which shifts cellular ratios of distinct Ub-chain types, resulting in autophagy or proteasomal activation. Interestingly, the reduced proteasome activity found in BTZ-resistant cells seems to be sufficient to permit the dTRIM24 PROTAC to function. In fact, when extracting the proteasomes from BTZ-resistant cell lines, their basal activity was always higher compared to the parental BTZ-sensitive cells. While the extraction from ZBR cells favors the recovery of more proteasomes compared to the parental cell line Z-138, this does not seem to be the case for JBR cells compared to the parental JeKo-1 cells. While this may be a direct consequence of the absence of active p53 [41], this has to be demonstrated. Could this be the reason why the BTZ/dTRIM24 combination treatment is more effective in JBR cells? This and other questions are to be answered in our future work.

Thus, our results indicate that the UPS-ALS crosstalk is not regulated in the same way in BTZ-resistant cells and that TRIM24 plays a major role in regulating the BTZ sensitivity. Together with proteaphagy, TRIM24 could be considered as a putative biomarker of BTZ resistance. More importantly, TRIM24 is a promising therapeutic target to recover sensitivity to BTZ. The results generated *in ovo* using xenografted ZBR cells support the role of TRIM24 in the regulation of the sensitivity to BTZ, and in the control of MCL metastatic properties toward secondary lymphoid tissues. Of importance, the absence of systemic toxicity during the administration of the drug combination in the two ZBR^TRIM24 WT^ and ZBR^TRIM24 KO^ *in ovo* models (data not shown) support the development of PROTAC-based strategies in treatment-resistant MCL. Further experiments are required using different BTZ-resistant cell lines and in vivo patient-derived *xenograft* no need to include xenograft models to expand our conclusions.

Although BTZ was approved more than a decade ago for the treatment of MCL and MM [42], the appearance of acquired or innate resistance in patients can limit its therapeutic potential. Multiple molecular mechanisms have been associated to BTZ resistance supporting a multifactorial origin of this phenotype. Chemical bromodomains inhibitors have been proposed, and some of them validated, as potential anti-cancer therapies, however without enough efficiency to validate their clinical relevance [43]. PROTACs have emerged as efficient degradation strategies to neutralize the activity of aberrantly expressed proteins [44–47]. The dTRIM24 used in this study supports the crucial role of this E3 enzyme in cancer development [26] and in particular to chemotherapy resistance in our MCL model. Further developments to delete optimize these therapeutic molecules may bring new single and combination therapies for cancer patients in years to come.

## Materials and methods

### Antibodies

Antibodies against GAPDH (Cell Signaling Technology Cat# 97166, RRID: AB_2756824), LC3B (Cell Signaling Technology Cat# 2775, RRID: AB_915950), p53 (Santa Cruz Biotechnology Cat# sc-126, RRID: AB_628082), PSMA2/α2 (Cell Signaling Technology Cat# 11864, RRID: AB_2797748), PSMB5/β5 (Cell Signaling Technology Cat# 12919, RRID: AB_2798061), PSMB6/ β1 (Enzo Life Sciences Cat# BML-PW8140, RRID: AB_10538396), PSMB7/ β2 (Santa Cruz Biotechnology Cat# sc-58410, RRID: AB_785340), PSMD4/ RPN10 (Invitrogen Cat# PA5-30136, RRID: AB_2547610), SQTM1/ p62 (Santa Cruz Biotechnology Cat# sc-28359, RRID:AB_628279), TRIM24 (Proteintech Cat# 14208-1-AP, RRID:AB_2256646), Tubulin (Sigma-Aldrich Cat# T3526, RRID:AB_261659) and ubiquitin (Santa Cruz Biotechnology Cat# sc-8017, RRID:AB_628423) were used. Peroxidase Goat Anti-Rabbit IgG (Jackson ImmunoResearch Labs Cat# 111-035-045, RRID:AB_2337938) and Peroxidase Rabbit Anti-Mouse IgG (Jackson ImmunoResearch Labs Cat# 315-035-003, RRID:AB_2340061) were used as secondary antibodies for WB assays.

### Cell culture

BTZ-resistant ZBR cell line was derived from Z-138 (BTZ-sensitive, WT-p53, WT-ATM) [30, 48] and was cultured in RPMI 1640 with 2 mM L-glutamine, 100 Units/mL penicillin, 100 µg/mL streptomycin and 10% fetal bovine serum (FBS), incubated at 37 °C, 5% CO_2_. BTZ-resistant JBR cell line was derived from JeKo-1 (BTZ-sensitive, p53 mut) [30, 48], and cultured in RPMI 1640 with 2 mM L-glutamine, 100 Units/mL penicillin, 100 µg/mL streptomycin and 20% fetal bovine serum (FBS), incubated at 37 °C, 5% CO_2_. HeLa cells were cultured at 37 °C and 5% CO_2_ in Dulbecco’s modified Eagle Medium (DMEM) supplemented with 10% fetal bovine serum (FBS, Gibco) and 1% penicillin/streptomycin (Gibco). Cells were treated with indicative doses of bortezomib (Tebu-Bio Cat# 27028), Bafilomycin A1 (Invivogen Cat# tlrl-baf1) or dTRIM24 PROTAC (MedChemExpress Cat# HY-111519). The absence of mycoplasma was routinely checked in all cultures by PCR and the identity of all cell lines was verified by using AmpFISTR identifier kit (Thermo Fisher).

### Western blotting

Mammalian cell extracts were lysed in 1.5X Boiling Buffer (BB) (4% SDS, 20% glycerol, 120 mM Tris-HCl pH 6.8). Proteins were separated loaded into a polyacrylamide gel, 120 V during 1 h, until the front of the gel disappears. Protein wet-transfer was performed onto PVDF or nitrocellulose membrane (0.45 µm pore size, Immobilon-P Merck Cat# IPVH00010) in buffer containing 20% ethanol and 80% 1X Transfer Buffer (10x: 240 mM TRIS, 2 M Glycine) for 1,5 h (335 mA per 2 gels at 4 °C).

Membranes were blocked for 1 h in 5% milk in Tris-buffered saline (TBS), then incubated with primary antibodies overnight at 4 °C. HRP Secondary antibodies were added for 1 h at RT in TBS. Membranes were washed 5 times, 5 min with TBS after incubation with primary and secondary antibodies. Pictures were acquired using West Femto ECL (Thermo Fisher Scientific Cat# 34096) with ChemiDoc Imaging System (BioRad), or iBrigh system (ThermoFisher Scientific). Quantifications were performed using ImageJ.

### Nuclear and cytoplasmic fractionation

The Thermo Fisher Scientific NE-PER™ Nuclear and Cytoplasmic Extraction (Thermo Fisher Scientific, Cat# 78833) protocol was followed for the separation of cytoplasmic and nuclear extracts from mammalian cultured cells (Z-138 and ZBR).

Cells were pelleted after 3 washes with PBS 1X. (8–10 × 10^6^ cells) by centrifugation at 500 × g for 2–3 min. Cytoplasmic Extraction Reagent (CER) I was added (200 μl), and vortexed vigorously for 15 s to fully resuspend the cells. Then cell extracts were incubated on ice for 10 min. CER II (11 μl) was added to the tubes, vortexed and incubated on ice for 1 min. Tubes were centrifuged for 5 min at maximum speed in a microcentrifuge (~16,000 × g). Supernatant was kept as the cytoplasmic extract (mixed with BB). Pellets were resuspended (insoluble fraction, nucleus) in cold Nuclear Extraction Reagent (NER) (50 μl). Tubes were kept on ice upon continue vortexing for 15 s every 10 min, for a total of 40 min. Samples were centrifuged at maximum speed (~16,000 × g) for 10 min and supernatant (nuclear extract) was kept mixed with BB and/or stored at -80 °C until use.

### Immunofluorescence microscopy

Immunofluorescence of Z-138 and ZBR cell lines was performed using glass slides (Immunocell Cat# 61.100.58) incubated 1 h at RT with poly-L-lysine 0.1% (w/v) in H_2_O (Sigma-Aldrich Cat# P8920). After that, cells were incubated on the glass slides in 1X PBS (1 million cells/mL), and fixed with 4% PFA, 10 min, and cold methanol 100% for 5 min. Samples were incubated in blocking solution (10% BSA, 0.1% Triton 100X in 1X PBS) 1 h at RT. Primary antibodies were incubated overnight, and secondary (1/500) antibodies 30 min, at RT in 5% BSA blocking solution. TRIM24 rabbit antibody was detected using Alexa 488 donkey anti-rabbit (green). p62 mouse antibody was detected using Alexa 568 donkey anti-mouse (red). Images were acquired using super-resolution microscopy Leica SP8 Lightning and assembled with Adobe Photoshop 7.0.

Immunofluorescence of HeLa cells was performed on 11 mm coverslip fixed in 4% PFA for 10–15 min, washed with PBS 1X, and permeabilised in 100% cold methanol for 3 min. Coverslips were washed 3 times with 1X PBS. Blocking was performed for 30 min at RT in Blocking Buffer (2% FBS, 1% of BSA and 1X PBS). Primary antibodies were incubated 1 h at 37°C, or 2 h at RT. Secondary antibodies (1:200) were incubated 30 min at 37°C, or 1 h at RT.

Nuclei were stained using DAPI (Invitrogen Cat# P36931). Images were acquired using Axio Imager D1 Zeiss Microscope, or super-resolution microscopy Leica SP8 Lightning and Zeiss LSM 880 Fast Airyscan and assembled with Adobe Photoshop 7.0. Images were not modified other than adjustments of levels, brightness, and magnification for a better visualization.

### Generation of ZBR^TRIM24 KO^ cell lines

CRISPR-Cas9 gene editing was used to generate ZBR^TRIM24 KO^ cells. Briefly, ZBR parental cells were resuspended in the Ingenio electroporation solution (Mirus Bio) and electroporated with SpCas9 Nuclease V3 and gRNA TRIM24/tracRNA ATTO 550 duplex (IDT-Integrated DNA Technologies) using a Nucleofector II device (program A032, Lonza). Single clones were obtained by a limiting dilution assay, checked for TRIM24 protein levels and genomic DNA was analysed by PCR and Sanger sequencing.

### Cell proliferation assays

Proliferation was determined by MTT [3-(4,5-dimethylthiazolyl-2)-2,5-diphenyltetrazolium bromide] assay (Sigma-Aldrich, Cat# 75989) to evaluate the effect of BTZ on the ZBR parental cells and on the ZBR^TRIM24 KO^ subclone after 24 h incubation. The IC_50_ was defined as the concentration required to reduce proliferation by 50% and was calculated with GraphPad Prism software (GraphPad Prism v10).

### Flow cytometry

Cells were collected from a 12- or 24-well plate, seeded at 4 × 10^5^ per mL, 1 day before treatment. Cells pelleted by centrifugation at 125 × *g* for 5 min, washed with 1X PBS, and resuspended with 1X Annexin Buffer, 1:100 FITC Annexin V and 1:100 Propidium Iodide (FITC Annexin V Apoptosis Detection Kit I BD Pharmingen™ Cat# 556547, RRID:AB_2869082). Three hundred μL of Annexin Buffer were added to samples, 10.000 events were acquired on BD Accuri C6, LSRFortessa or MACSQuant VYB and analyzed using FlowJo software. Mean and standard deviation of all events were calculated from the population.,

Changes in mitochondrial transmembrane potential (ΔΨm) were assessed by staining cells with 20 nM 3,3′-diexyloxacarbocyanine iodide (DiOC6) (Thermo Fischer Scientific Cat# D273) for 15 min at 37 °C. In both analyses, 10.000 labeled cells per sample were acquired on a FACS CantoII flow cytometer (Becton Dickinson, San Jose, CA, USA) and analyzed using FlowJo software.

### Chicken embryo chorioallantoic membrane (CAM) assay

Fertilized white Leghorn chicken eggs were purchased from Granja Santa Isabel, S. L. (Córdoba, Spain) and incubated for 9 days at 37 °C with 55% humidity. At day 9 of their embryonic development (ED9), eggs were cleaned with ethanol 70° and a window of an ~2 cm-diameter was drilled on top of the air chamber of the eggshell. Then, one million ZBR^TRIM24 WT^ or ZBR^TRIM24 KO^ cells per egg were resuspended in 25 µL RPMI medium containing 10% FBS and 100 U/mL penicillin and streptomycin (Thermo Fisher) and 25 µL Matrigel (BD Biosciences). The mix was incubated for 15 min at 37 °C and subsequently implanted into the CAM. The window was then covered with a sterile tape and the eggs were placed back in the incubator. At ED12 and ED14, 5 nM BTZ, 10 µM dTRIM24, the combination of both, or vehicle, all diluted in complete RPMI medium were administered topically on 8–10 tumor-bearing CAMs. At ED16, chick embryos were sacrificed by decapitation. Tumors were excised and carefully weighed to determine their mass. DNA from *n* = 3 representative tumor specimens and chick embryo’s spleen was isolated according to manufacturer instructions using a Genomic DNA Purification Kit (Promega). The metastasis rate was evaluated by quantitative real-time PCR; the relative amount of human Alu sequences was quantified using the comparative cycle threshold method (ΔCt) as previously described [31].

### Quantification and statistical analysis

Quantification and statistical analysis were performed using three independent experiments (biological replicates), with all points normalised to the control sample (No treatment, NT).

Two-way ANOVA (post-hoc test) with multiple comparisons, or *t*-test student analysis were applied for comparisons between conditions using the GraphPad software for statistical analysis and visualization. The data are presented as the means ± SEM. The ∗*p* < 0.05, ∗∗*p* < 0.01 or ∗∗∗*p* < 0.001 values were considered statistically significant.

Combination index (CI) was calculated using the Compusyn software (http://www.combosyn.com). CI < 0.8 indicates synergistic effect. 0.8 < CI < 1.2 indicates additive effect and CI > 1.2 indicates antagonism effect.

### Native gel electrophoresis and proteasome activity assay

*In gel* chymotrypsin-like activity of the proteasome β5 subunit was measured in vitro from MCL cells using reporter peptides [24, 49]. Five million rapidly thawed cells were used for each experimental point. 30 μg of total protein were migrated per well in NuPAGE™ Novex™ 3–8% Tris-Acetate Protein Gel (Thermo Fisher Scientific Cat# EA0375PK2). Migrations were performed in native gel electrophoresis buffer (NG buffer) (90 mM Tris-borate, 0.1 mM EDTA, 5 mM MgCl_2_, 0.5 mM ATP, 0.5 mM DTT) at 150 V for 3 h. The intrinsic activity of native proteasomes was analysed in gel by 20 min incubation in NG buffer supplemented with 100 µM Suc-LLVY-AMC (Bachem Cat# 4011369) at 37 °C. The amount of cleaved AMC fragment was imaged with Syngene NuGenius. Native gels were then washed twice for 10 min in 10X Tris-glycine–SDS Laemmli buffer (0.25 M Tris, 1.92 M glycine, 1% SDS, pH 8.6), followed by a final wash in 1X Tris-glycine–SDS Laemmli buffer. Gels were transferred in PVDF membranes (0.45 µm pore size, Immobilon-P Merck Cat# IPVH00010) overnight at 40 V at 4 °C. Membranes were blotted to detect the proteins of interest.

### Nanobodies pull-down (Nanobodies precipitations NP)

10–20 × 10^6^ cells were used in each condition along with 150 µg of each K48 or K63 nanobody (Nanotag Biotech). Ub chain-specific Nanobodies were preincubated with Ni-NTA agarose-beads for 1 h rotating at 4 °C as reported [50]. Centrifugation steps (for washing the beads) are made at 1200 rpm, 3–5 min. 320 µL of dry beads, were used per sample point, incubated with 150 µg of Chain-Specific Nanobodies. Cells were lysed in 500 µL (each cell pellet of 10–20 × 10^6^ cells cells) of Lysis Buffer (50 mM sodium fluoride, 5 mM tetra-sodium pyrophosphate, 10 mM β-glyceropyrophosphate, 1% Igepal, 2 mM EDTA, 20 mM NaH^2^PO^4^, 1 mM Pefablock, 1.2 mg/mL), a complete protease inhibitor cocktail (Mini, EDTA-free Protease Inhibitor Cocktail-Roche, Cat# 04693159001), 10 μM PMSF, 10 mM N-ethylmaleimide (Thermo Fisher Scientific Cat# 23030) and 10 mM Iodoacetamide (Sigma Aldrich Cat# I1149) on ice for 10 min. Tubes were centrifuged at 13,000 rpm for 10 min at 4 °C, and the supernatants were incubated with the beads, rotating at 4°C for overnight after adding 200 mM of NaCl. Washing steps were made as follows: 3 washes PBS 1X-Tween 0.1% 300 mM NaCl, 2 washes PBS 1X-Tween 0.05%, and 2 washes PBS 1X. After the washes, beads were resuspended in 100 µL of BB and heated 45 min 95 °C with strong agitation.

## Supplementary information

Supplementary material

Supplementary Material Original Werstern blots

## Data Availability

The materials described in the manuscript, including all relevant raw data, will be freely available to any researcher wishing to use them for non-commercial purposes, without breaching participant confidentiality.
