# Supplementary material for: Role of TRIM24 in the regulation of proteasome-autophagy crosstalk in bortezomib-resistant mantle cell lymphoma
Source: Cell Death Discov. 2025 Mar 17;11:108. doi: 10.1038/s41420-025-02355-6 (PMC11914149; doi:10.1038/s41420-025-02355-6)
Supplement: Supplementary file 1 — Supplementary material [file 41420_2025_2355_MOESM1_ESM.pdf]

Supplementary Material

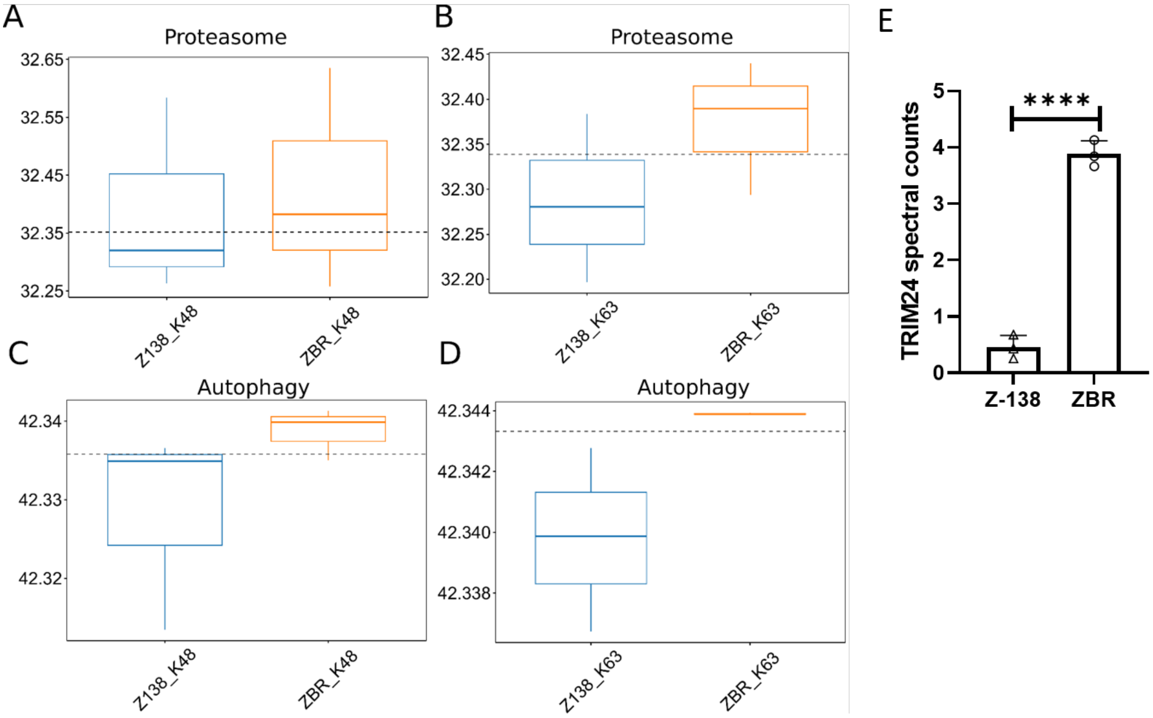

Supplementary Figure 1: The ubiquitin proteome associated to K63 chains and TRIM24 is enriched in BTZ resistant ZBR cells. A-D) K48 or K63 chain specific nanobodies were used to identify by mass spectrometry the ubiquitin proteome of BTZ-sensitive Z138 cells or BTZ-resistant ZBR cells. Overall abundance of autophagy and proteasome factors based on ion counts. Ion counts were estimated from pull down of K48 chains "A & C" and K63 chains "B & D". B, D) Proteasome and autophagy factors associated to K63 ubiquitin chains were found enriched in ZBR cells compared to K63 chains found in Z138 cells. E)TRIM24 was found enriched in the TUBE-associated ubiquitin proteome of ZBR cells compared to Z-138 cells (34)
.

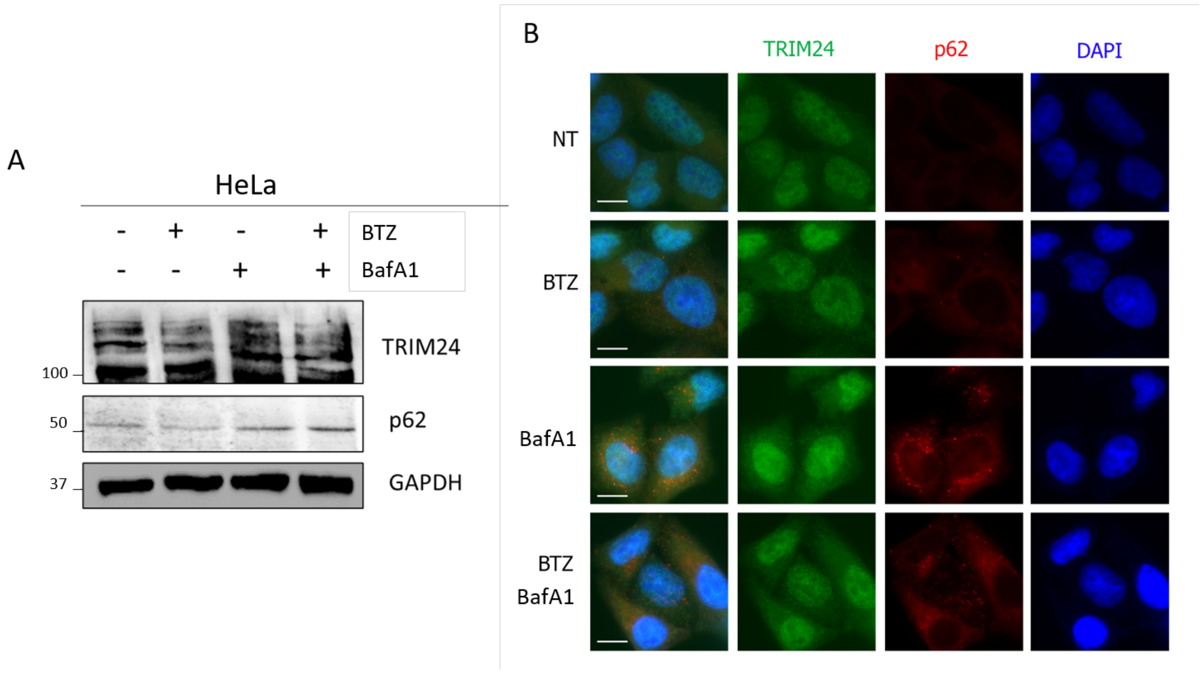

Supplementary Figure 2: TRIM24 protein levels are increased upon BafA1, and BTZ/BafA1 treatment in HeLa cells. A) HeLa cells treated with BTZ 40 nM, BafA1 5 μM, and the combination of both agents for 4 h. WB analyses to detect TRIM24, p62 and GAPDH levels. B) Immunofluorescence images of HeLa cells treated with BTZ 40 nM, BafA1 5 μM or the treatment combination for 4 h, using TRIM24 and p62 antibodies. TRIM24 rabbit antibody was detected with Alexa 488 donkey anti-rabbit (Green). p62 anti-mouse antibody was detected using Alexa 568 donkey anti-mouse (Red). Images were analysed using Axio Imager D1 Zeiss Microscope and assembled with Adobe Photoshop 7.0 (Scale bar: 10 μm).

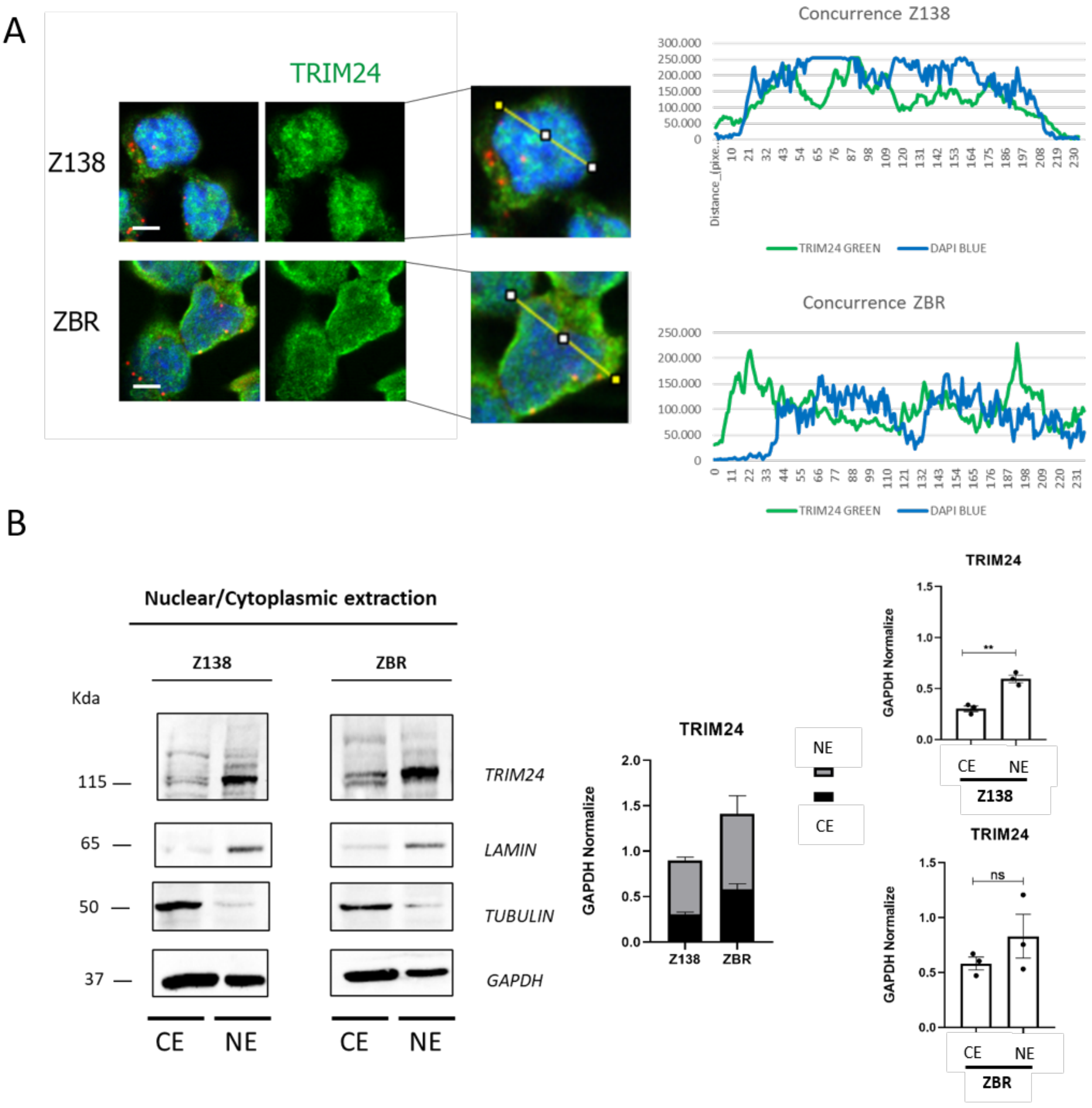

Supplementary Figure 3: Partial cytoplasmic localization of TRIM24 in ZBR cells. A) Immunofluorescence images show the localization of TRIM24 in Z-138 and ZBR cell lines under basal conditions (no treatment). TRIM24 was detected (green) with a rabbit antibody and an Alexa 488 donkey anti-rabbit antibody. DAPI stains the nucleus in blue. The right part of the panel shows concurrence analysis (Image J software) and displays TRIM24 distribution inside the nucleus when it overlaps with DAPI. Images were acquired using super-resolution microscopy Leica SP8 Lightning and assembled with Adobe Photoshop 7.0 (Scale bar: 10um). B) WB analysis of TRIM24 cytoplasmic/nuclear fractionation of Z-138 and ZBR cells. Soluble (C, Cytoplasmic), and Insoluble (N, Nuclear) fractions were obtained following instructions of

the manufacturer. Lamin antibody was used to control nuclear enrichment, and Tubulin antibody was used to control the cytoplasmic fractionation. Quantification of TRIM24 using GAPDH from the soluble fraction to normalize values. Graphics were made using GraphPad Prism Software. Data from the N and C fractions were included in an additional graphic for a better visualization of N/C distribution in each cell line. (n≥3; mean ± SD; two-way t-test , *p<0.05, **p<0.01, p***<0.001).

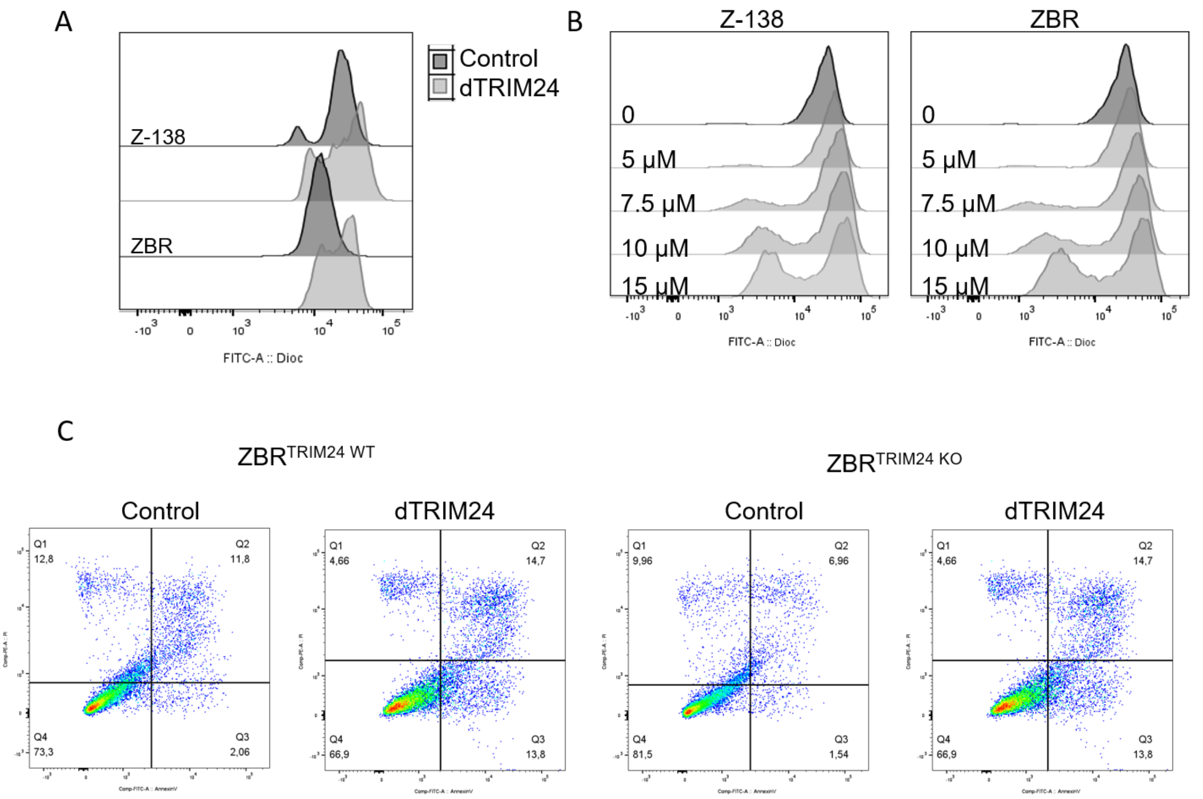

Supplementary Figure 4: Degradation of TRIM24 triggers cell death in both Z-138 and ZBR cell lines. Representative flow cytometry dot plots/histograms from Figure 3 for mitochondrial transmembrane potential staining experiments (A and B) annexin V (C).

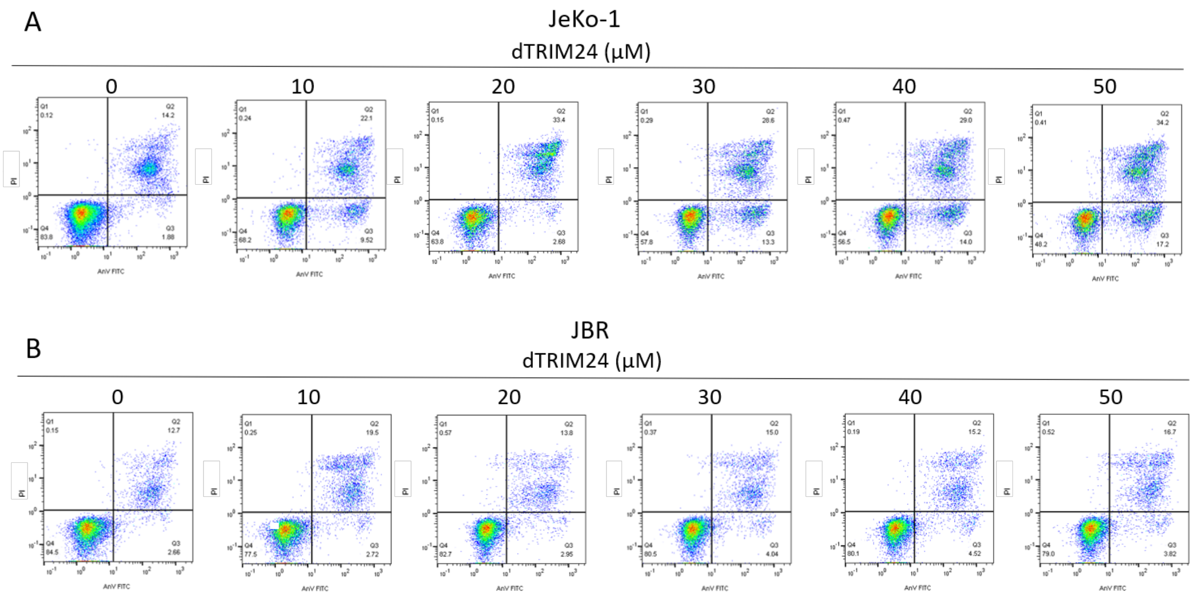

Supplementary Figure 5: dTRIM24 requires a fully functional proteasome but not p53 to efficiently drive apoptosis. Representative flow cytometry dot plots/histograms from Figure 4.

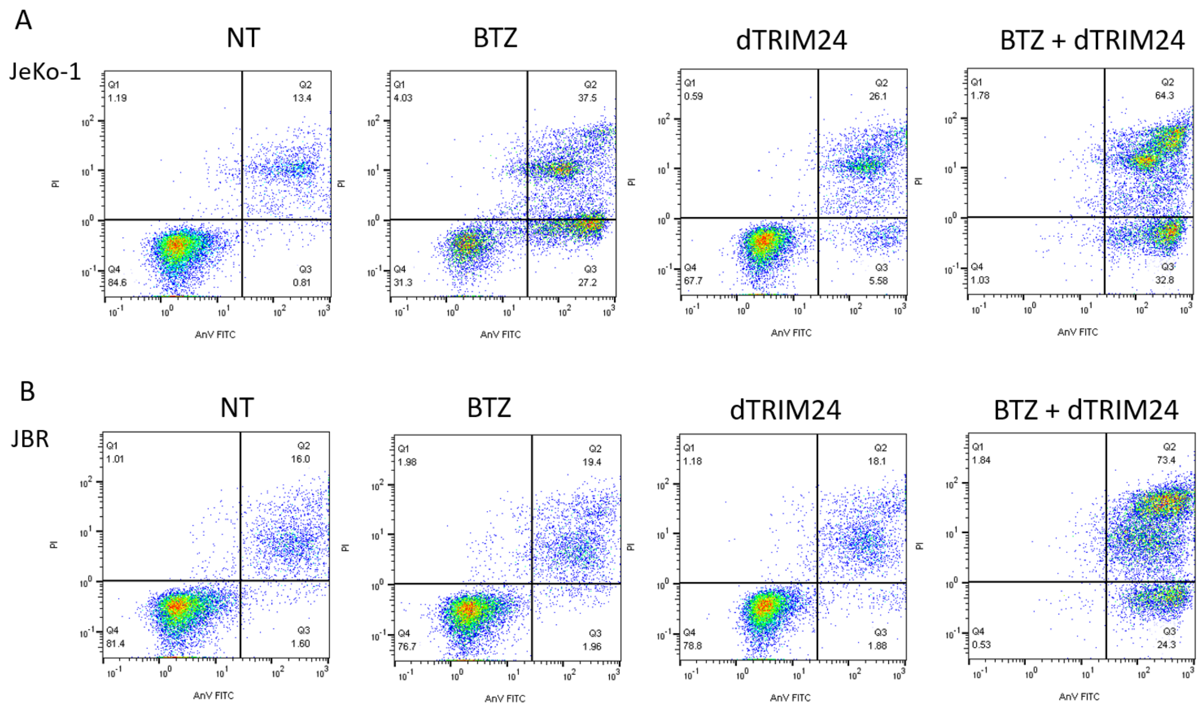

Supplementary Figure 6: Reduction of TRIM24 levels recover BTZ sensitivity in resistant cells. Representative flow cytometry dot plots/histograms from Figure 5.

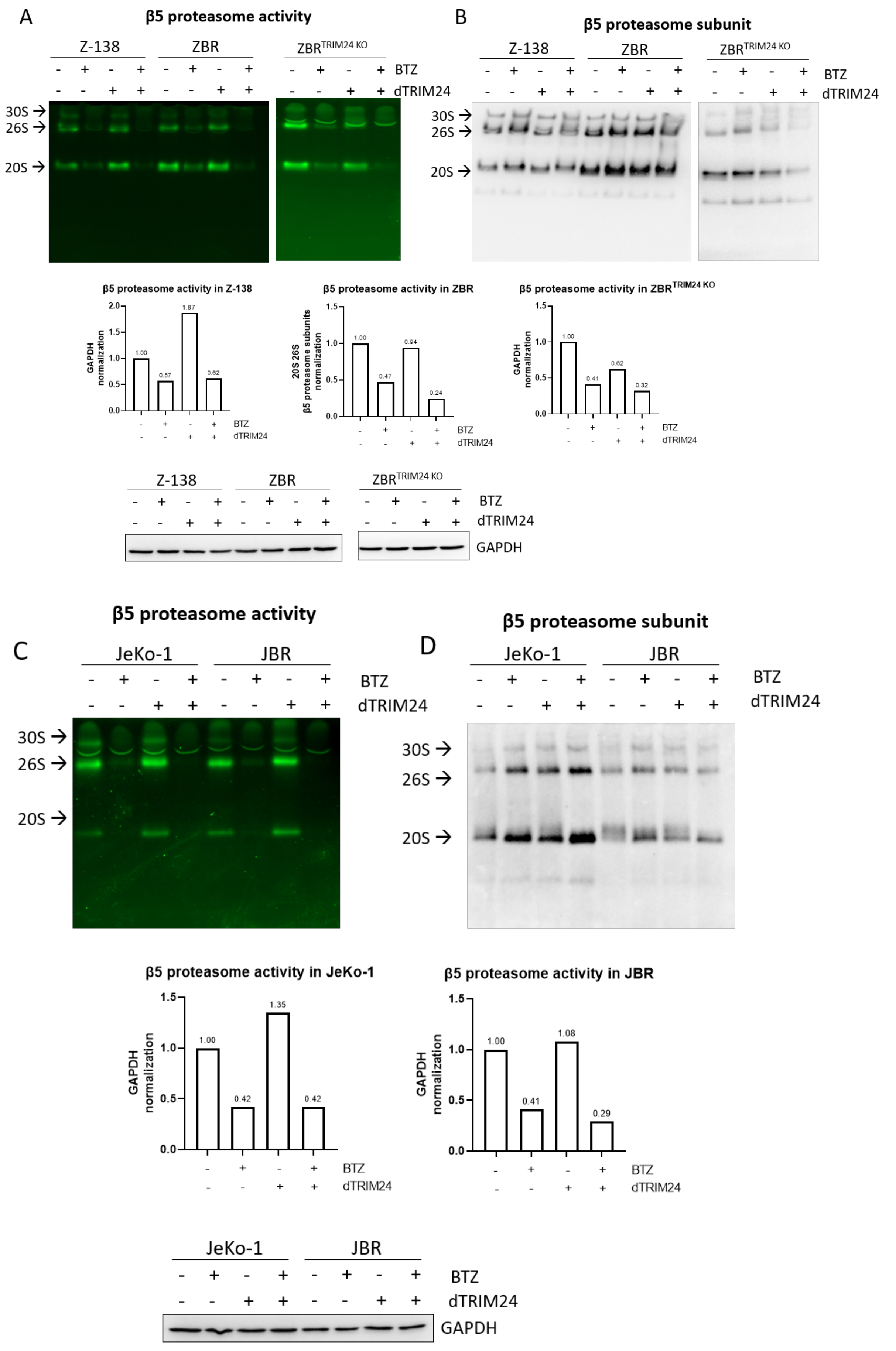
A
β5 proteasome activity
Z-138
ZBR
ZBR$^{TRIM24\ KO}$
BTZ
dTRIM24
30S
26S
20S
B
β5 proteasome subunit
Z-138
ZBR
ZBR$^{TRIM24\ KO}$
BTZ
dTRIM24
30S
26S
20S
β5 proteasome activity in Z-138
GAPDH normalization
1.00
0.57
1.87
0.62
BTZ
dTRIM24
β5 proteasome activity in ZBR
20S 26S β5 proteasome subunits normalization
1.00
0.47
0.94
0.24
BTZ
dTRIM24
β5 proteasome activity in ZBR$^{TRIM24\ KO}$
GAPDH normalization
1.00
0.41
0.62
0.32
BTZ
dTRIM24
Z-138
ZBR
ZBR$^{TRIM24\ KO}$
BTZ
dTRIM24
GAPDH
β5 proteasome activity
C
JeKo-1
JBR
BTZ
dTRIM24
30S
26S
20S
β5 proteasome subunit
D
JeKo-1
JBR
BTZ
dTRIM24
30S
26S
20S
β5 proteasome activity in JeKo-1
GAPDH normalization
1.00
0.42
1.35
0.42
BTZ
dTRIM24
β5 proteasome activity in JBR
GAPDH normalization
1.00
0.41
1.08
0.29
BTZ
dTRIM24
JeKo-1
JBR
BTZ
dTRIM24
GAPDH

Supplementary Figure 7: dTRIM24 treatment enhances proteasome activity. A) In-gel proteasome activity assays were performed with extracts of Z-138, ZBR and $ZBR^{TRIM24\ KO}$ cells treated or not with dTRIM24. The specificity of the fluorescent reporter peptide was assessed using cell extracts treated BTZ. Proteasomal activity was normalised using GAPDH detected by Western blot. B) The same gel used in "A" was western-blotted to detect the β5 proteasome subunit. 20S, 26S and 30S complexes as indicated. C) In-gel proteasome activity assays were performed with extracts of JeKo-1 and JBR cells treated or not with dTRIM24. The specificity of the fluorescent reported peptide was assessed using cell extracts treated with BTZ. Proteasomal activity was normalised using GAPDH detected by Western blot. D) The same gel used in "A" was western-blotted to detect the β5 proteasome subunit. 20S, 26S and 30S complexes as indicated.

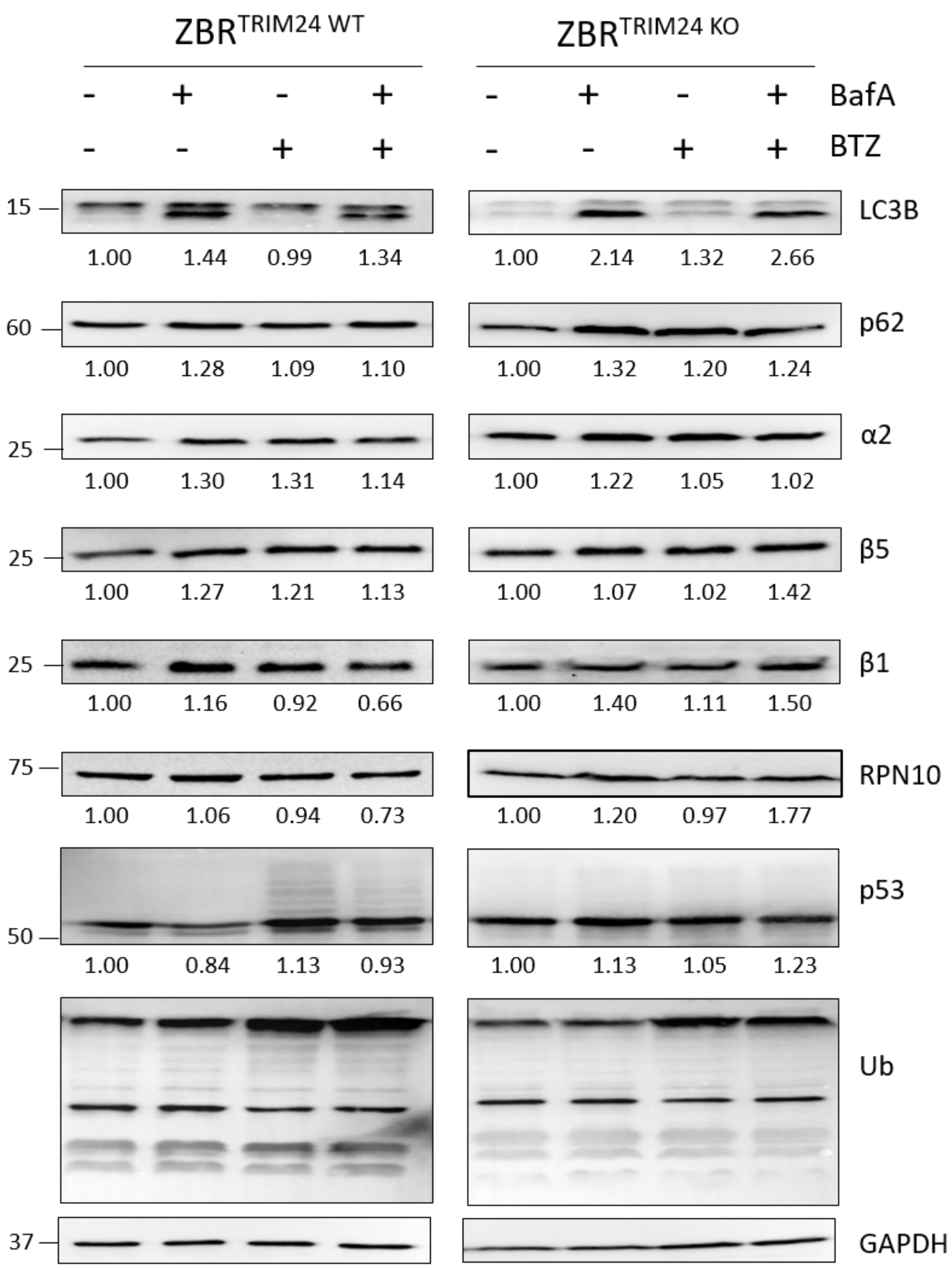

Supplementary Figure 8: TRIM24 does not directly regulate proteaphagy in ZBR cells. ZBR and ZBR$^{TRIM24\ KO}$cell lines were treated with 20 nM BafA1, 20 µM BTZ or the combined treatment for 6 h. Western blot analyses were carried out to detect LC3B, p62, a2, β5, β1 and RPN10 proteasomal subunits, total ubiquitination and p53. Quantifications were performed using ImageJ software (n≥3) and normalised against GAPDH values. Ratios changes were calculated using untreated conditions as control (1fold).

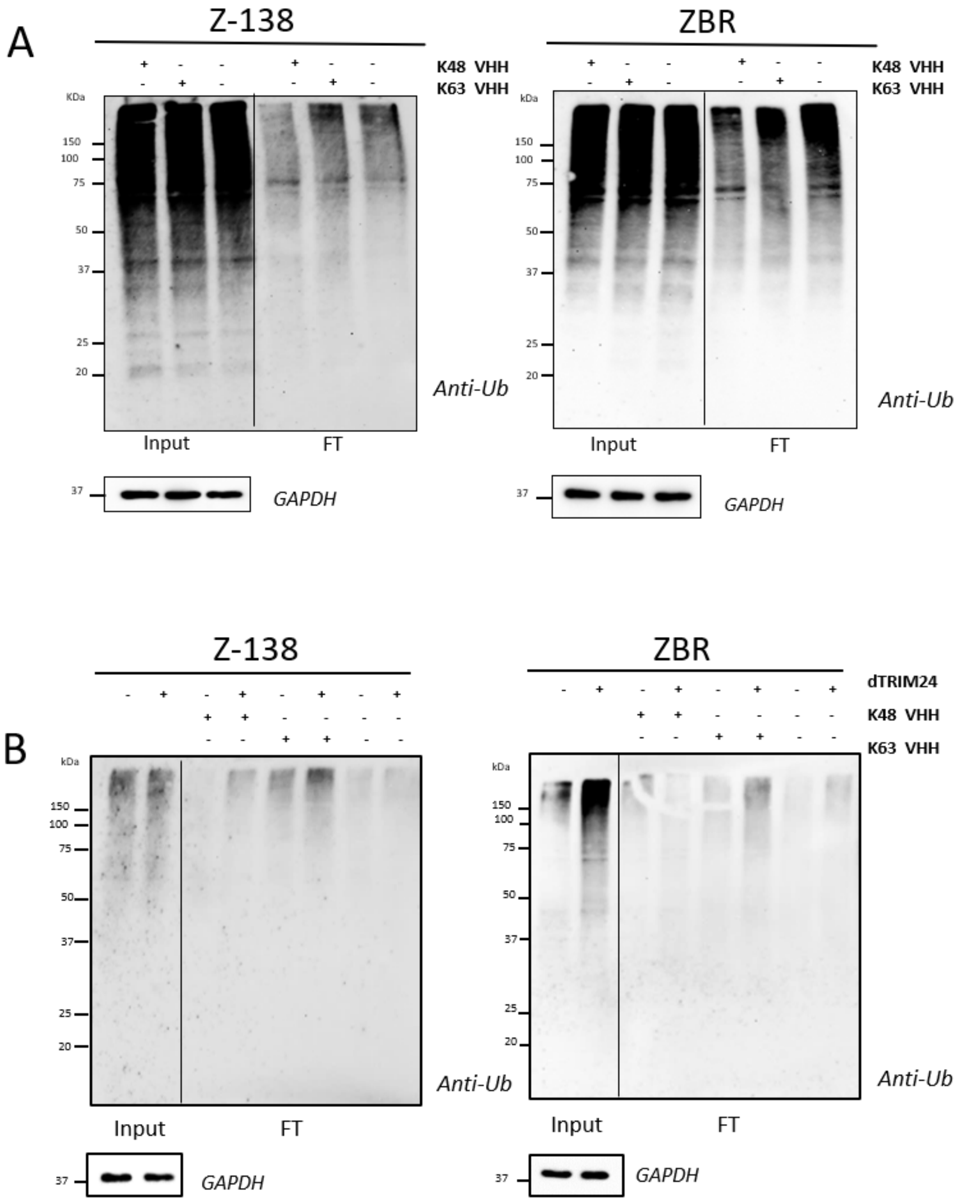

Supplementary Figure 9: Input and flow-through fractions of pulldowns performed with K48 and K63 chain specific nanobodies. Input and flow-through fractions of pull-down assays performed using K48 or K63 ubiquitin chain-specific nanobodies from Z-138 and ZBR. A) Levels of ubiquitylation at basal conditions were detected by Western blot using anti-Ub from the input, flow through (FT) fractions. GAPDH was used as loading control. A) Levels of ubiquitylation of cells treated or not with dTRIM24 were detected by Western blot using anti-Ub from the input, flow through (FT) fractions. GAPDH was used as loading control.
