# Supplementary material for: Role of TRIM24 in the regulation of proteasome-autophagy crosstalk in bortezomib-resistant mantle cell lymphoma
Source: Cell Death Discov. 2025 Mar 17;11:108. doi: 10.1038/s41420-025-02355-6 (PMC11914149; doi:10.1038/s41420-025-02355-6)

Title: Role of TRIM24 in the regulation of the proteasome-autophagy crosstalk in bortezomib resistant Mantle Cell Lymphoma cells.

Corentin Bouvier, Maria Gonzalez-Santamarta, Núria Profitós-Pelejà, Marc Armengol, Grégoire Quinet, Quentin Alasseur, Laurie Ceccato, Wendy Xolalpa, Raimundo Freire, Julie Guillermet-Guibert, Karine Reybier, Anne Marie Caminade, Hans C Beck, Ana Sofia Carvcalho, Rune Matthiesen, Jean Christophe Rain, James D. Sutherland, Rosa Barrio, Gaël Roué and Manuel S. Rodriguez.

Original western blots

Figure 1:

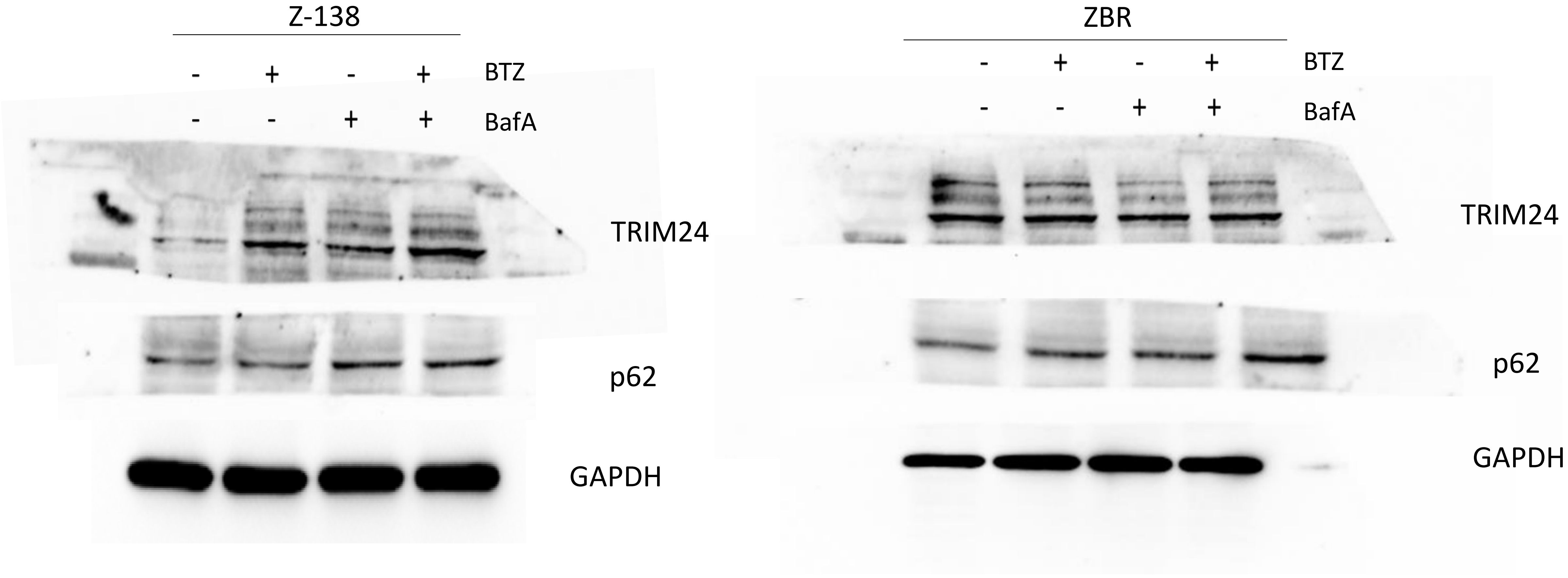

Figure 2:

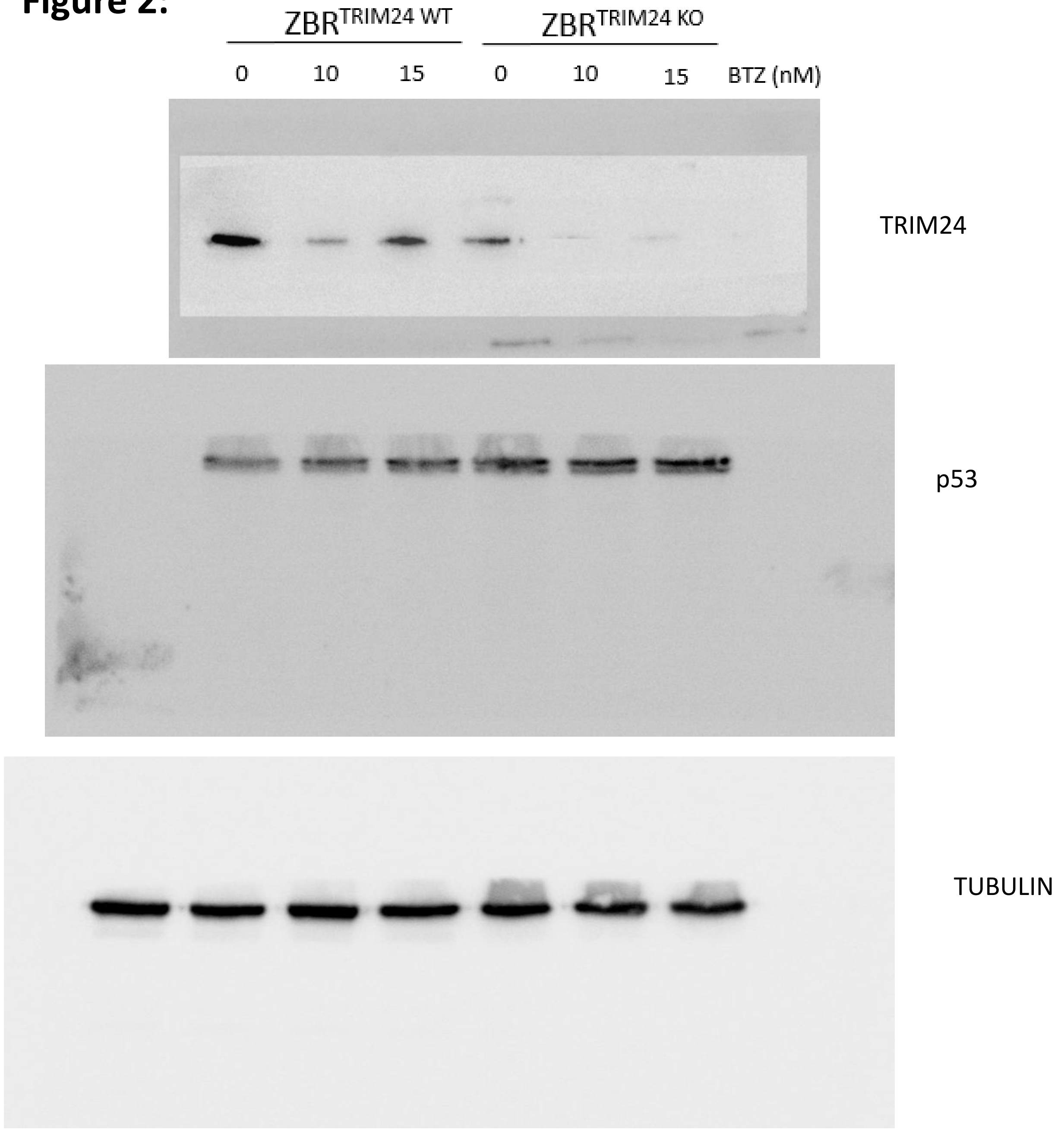

$ZBR^{TRIM24\ WT}$
$ZBR^{TRIM24\ KO}$
0 10 15 0 10 15 BTZ (nM)
TRIM24
p53
TUBULIN

Figure 3:

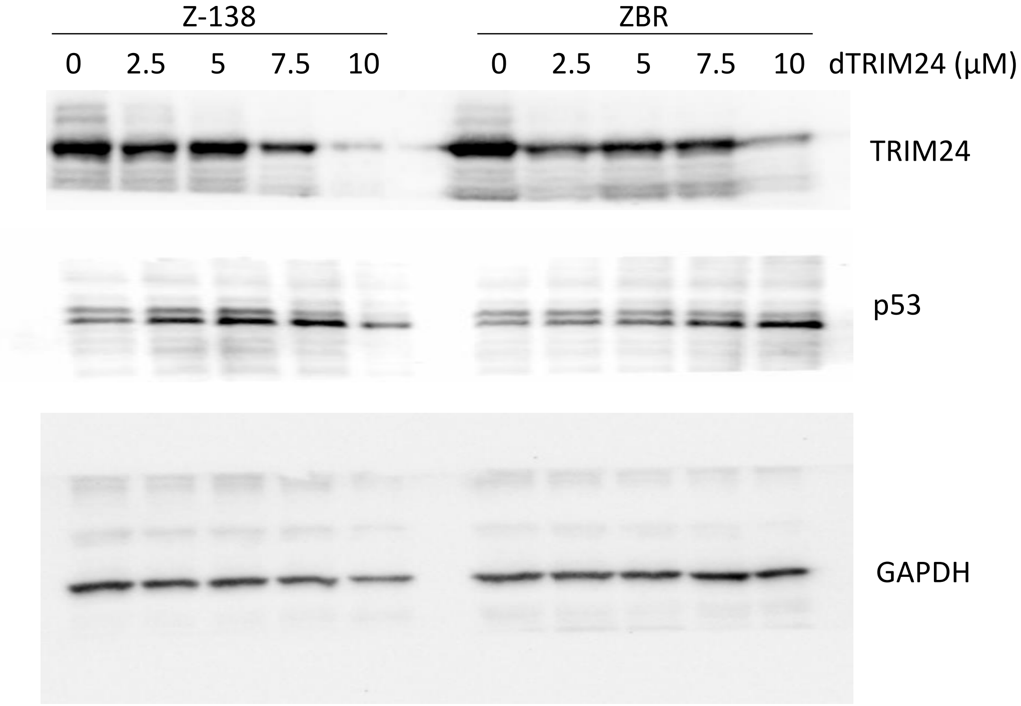

Figure 4:

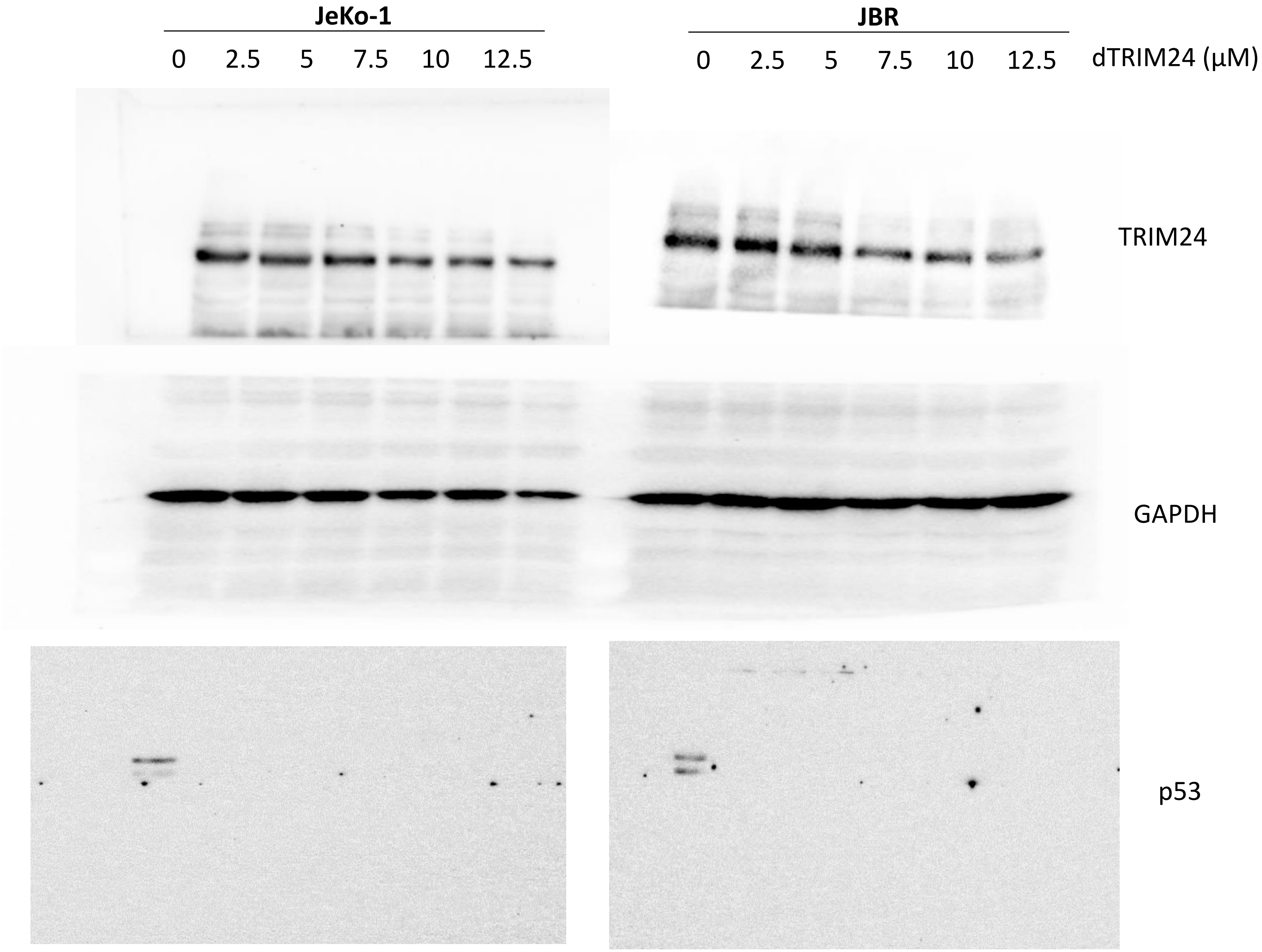
JeKo-1
JBR
0
2.5
5
7.5
10
12.5
0
2.5
5
7.5
10
12.5
dTRIM24 (µM)
TRIM24
GAPDH
p53

Figure 6:

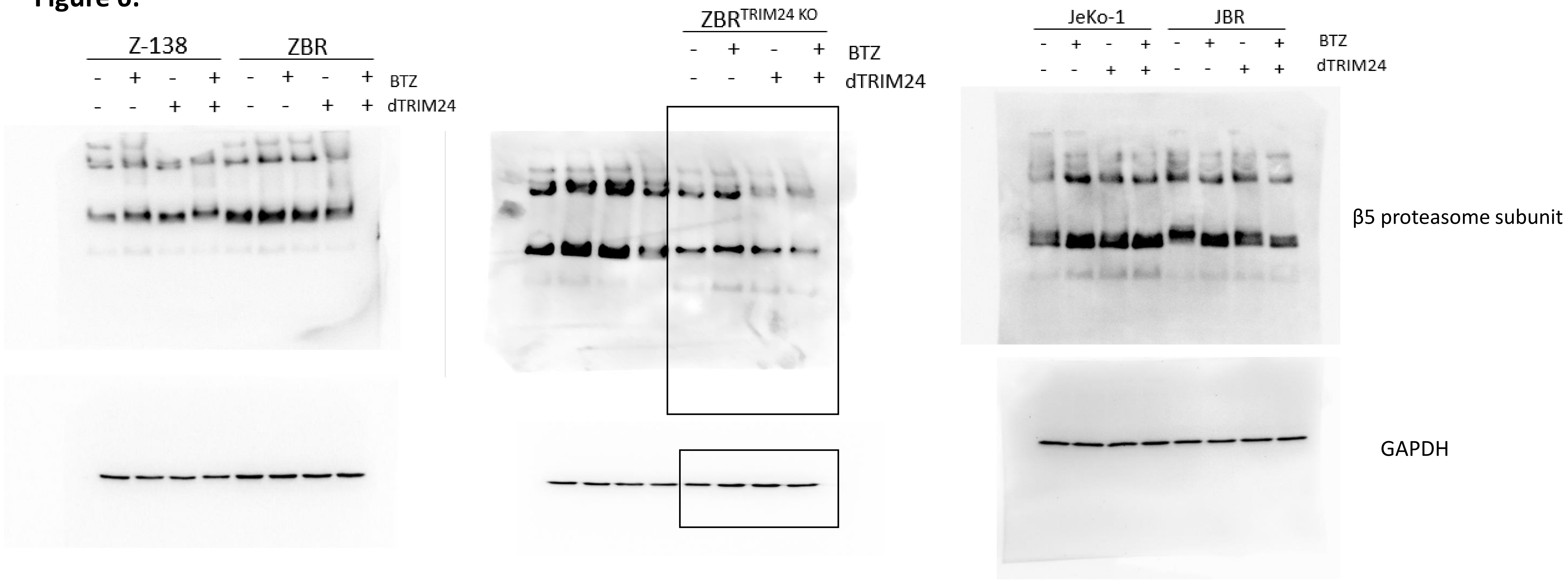
Z-138
ZBR
BTZ
dTRIM24
ZBR[TRIM24 KO]
BTZ
dTRIM24
JeKo-1
JBR
BTZ
dTRIM24
β5 proteasome subunit
GAPDH

Figure 7:

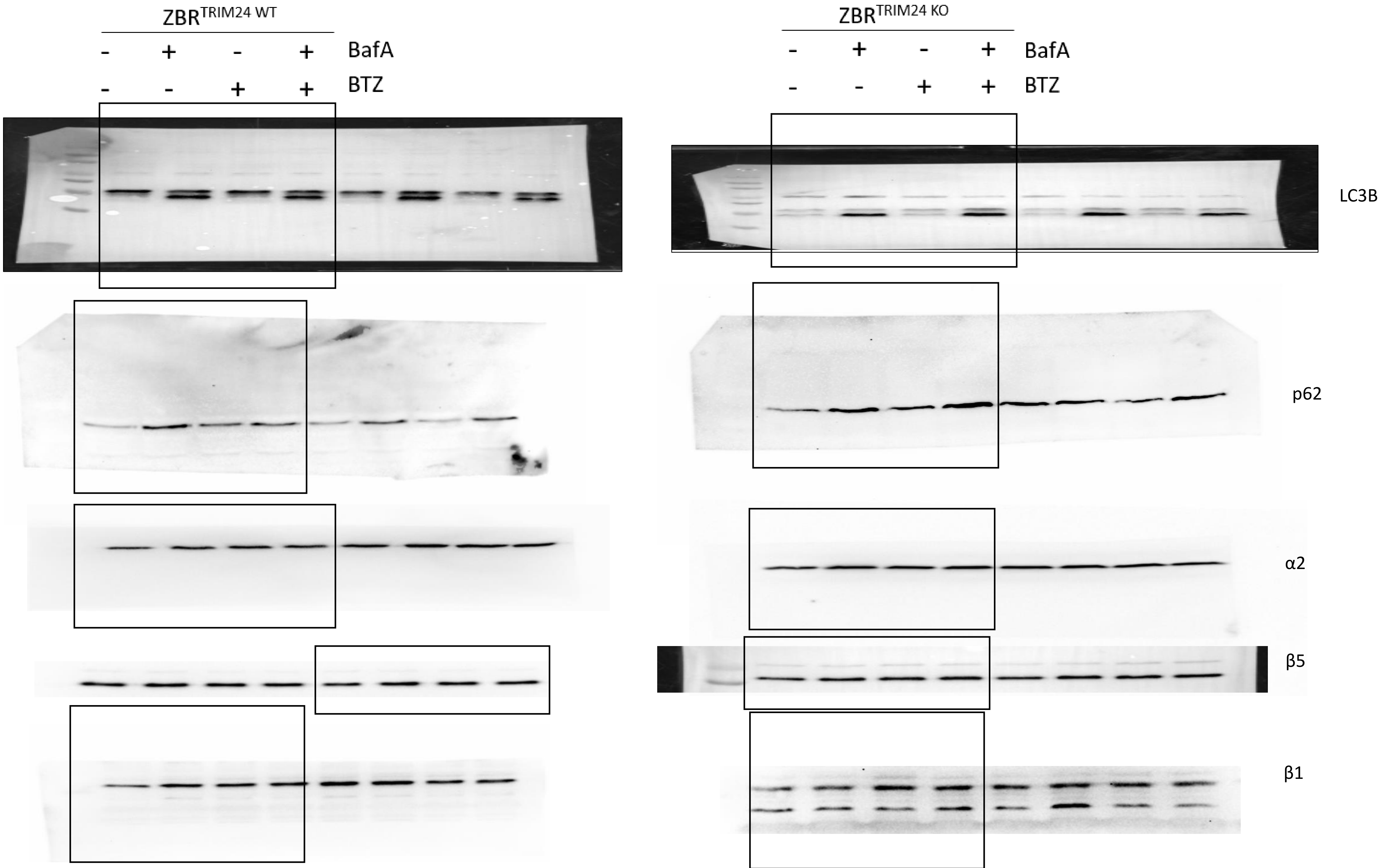

Figure 7:

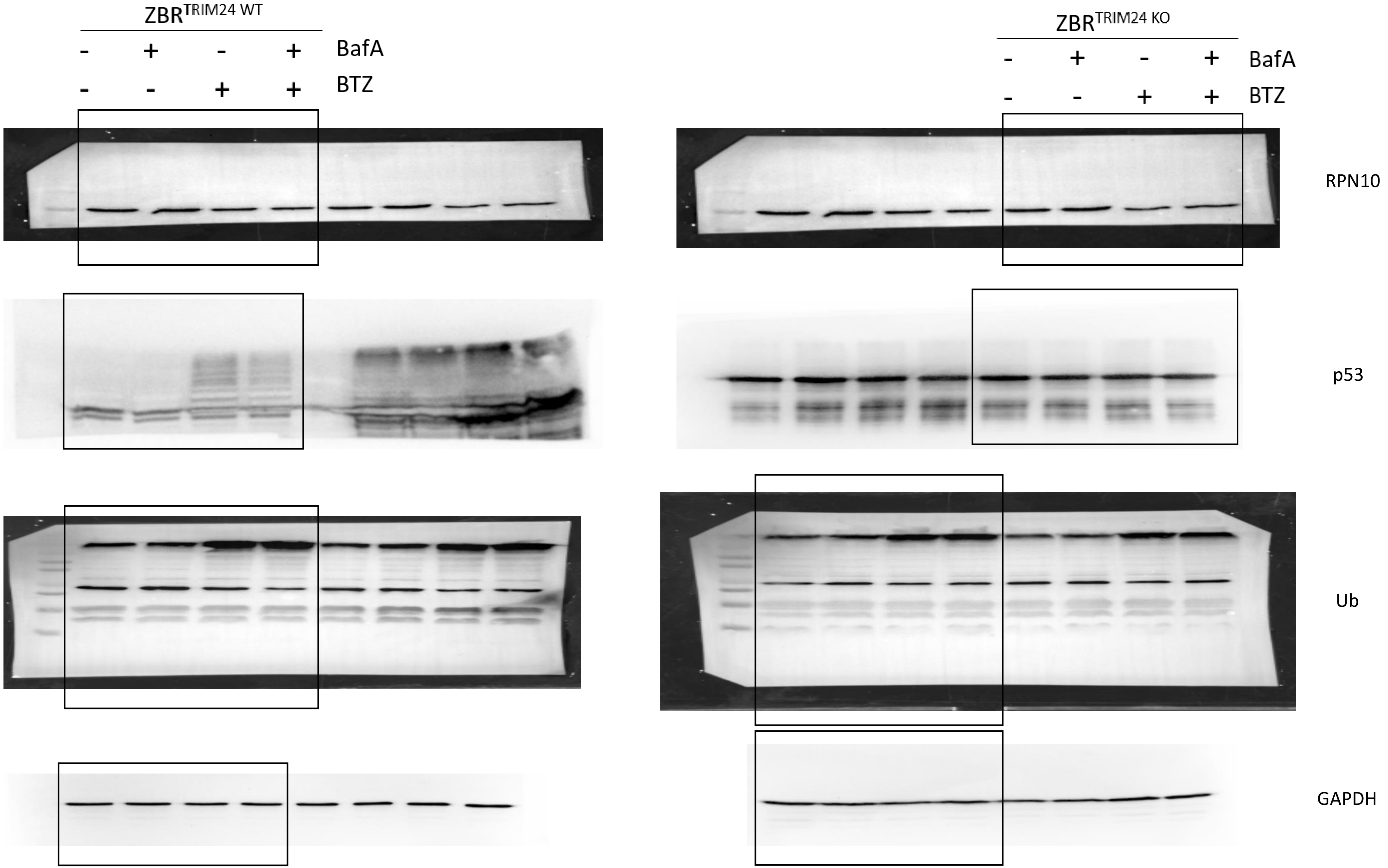
ZBR^TRIM24 WT
- + - + BafA
- - + + BTZ
ZBR^TRIM24 KO
- + - + BafA
- - + + BTZ
RPN10
p53
Ub
GAPDH

Figure 8:

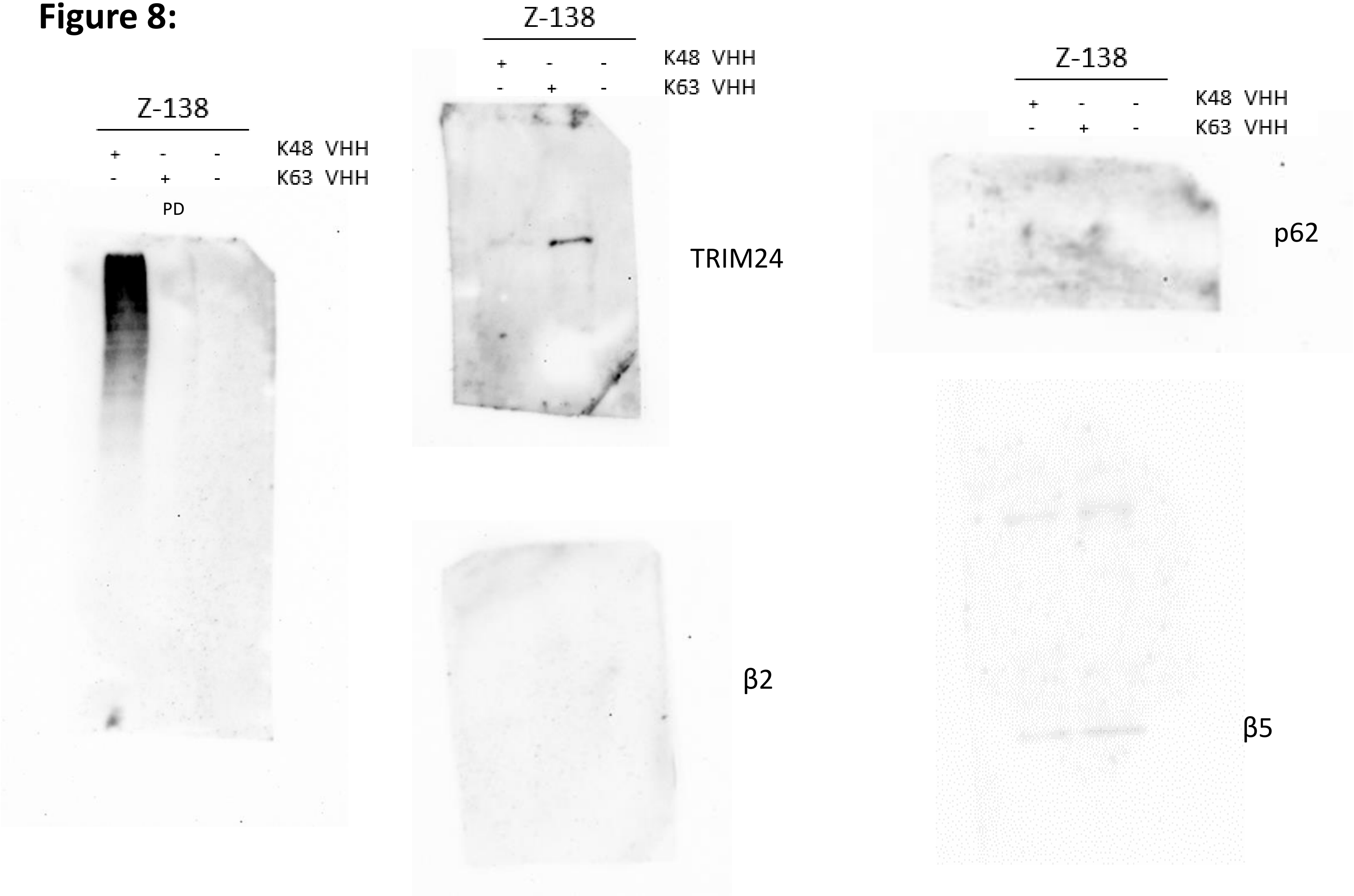

Figure 8:

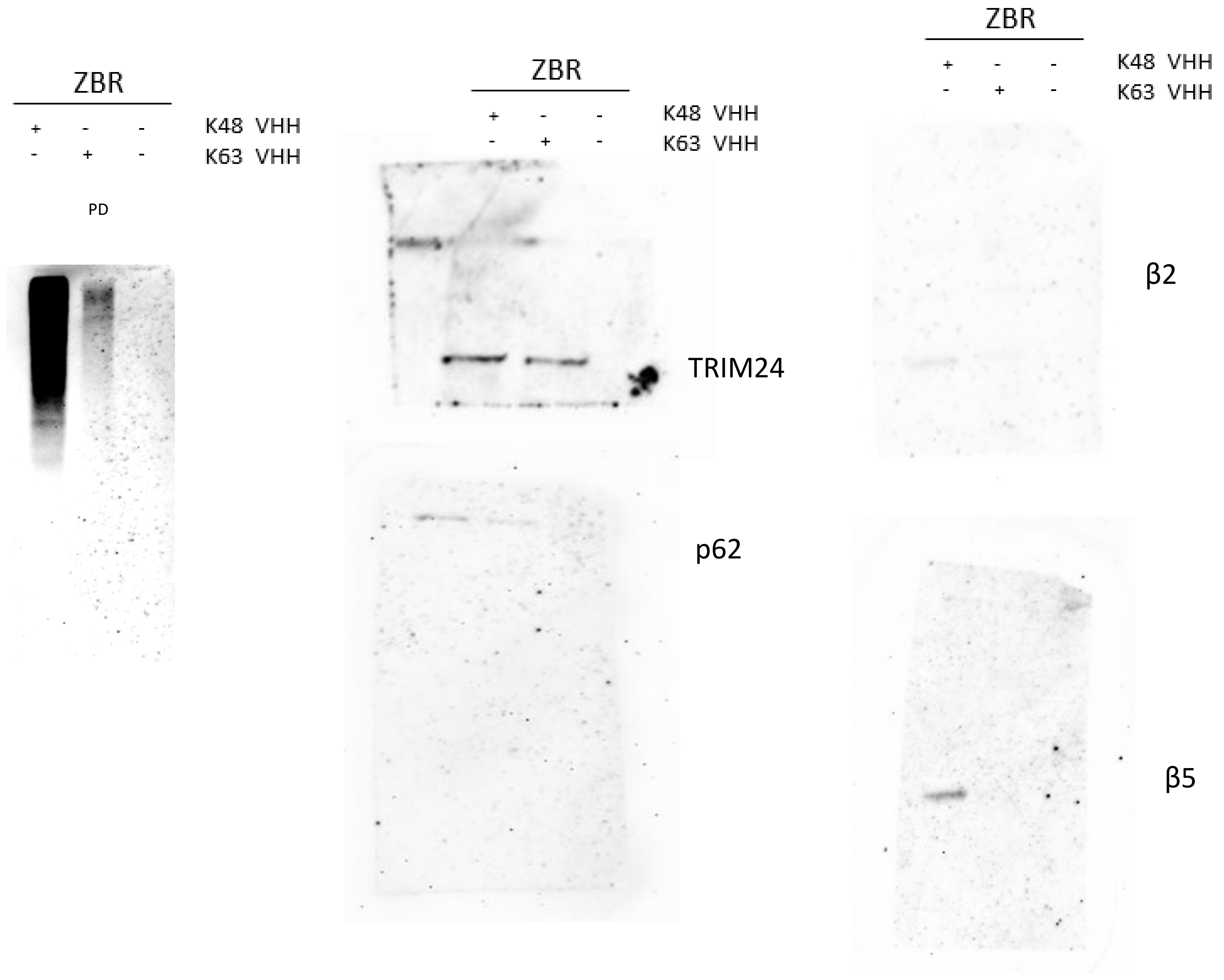
ZBR
+ - -
- + -
K48 VHH
K63 VHH
PD
ZBR
+ - -
- + -
K48 VHH
K63 VHH
TRIM24
p62
ZBR
+ - -
- + -
K48 VHH
K63 VHH
β2
β5

Figure 8:

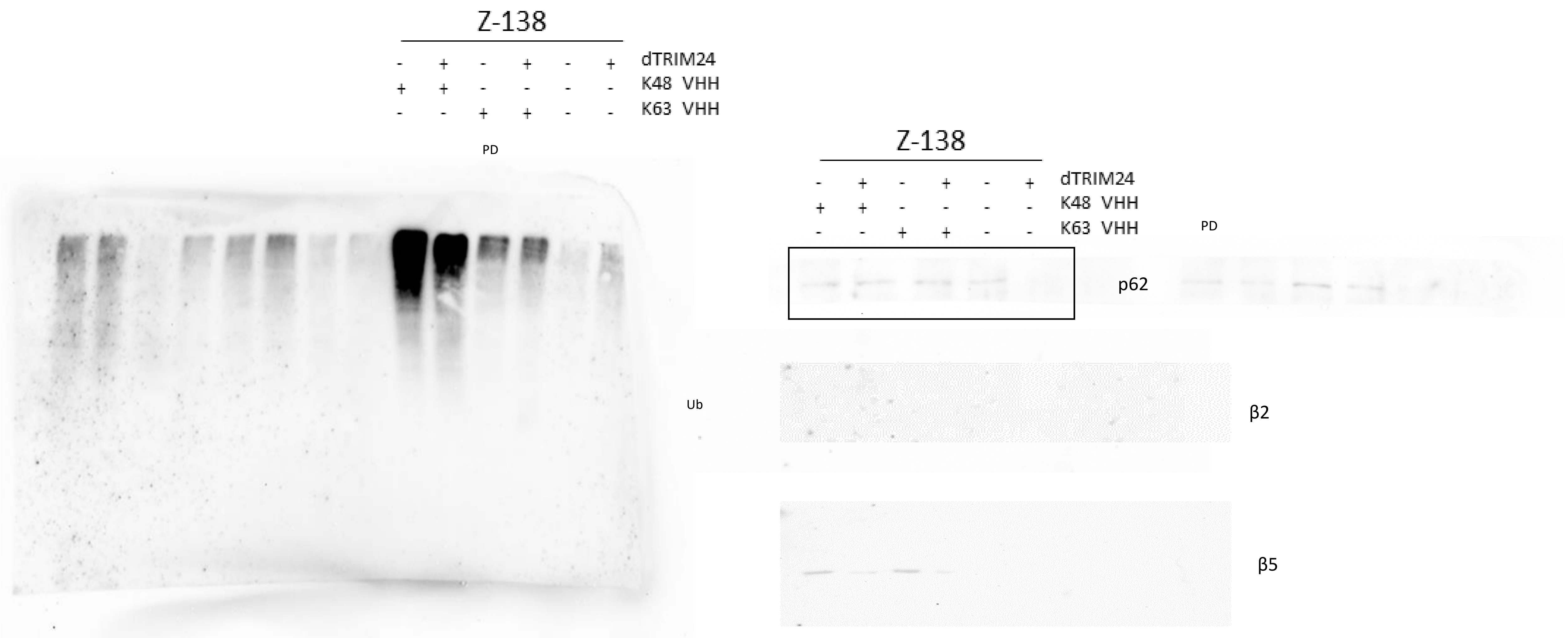
Z-138
dTRIM24
K48 VHH
K63 VHH
PD
Ub
Z-138
dTRIM24
K48 VHH
K63 VHH
PD
p62
β2
β5

Figure 8:

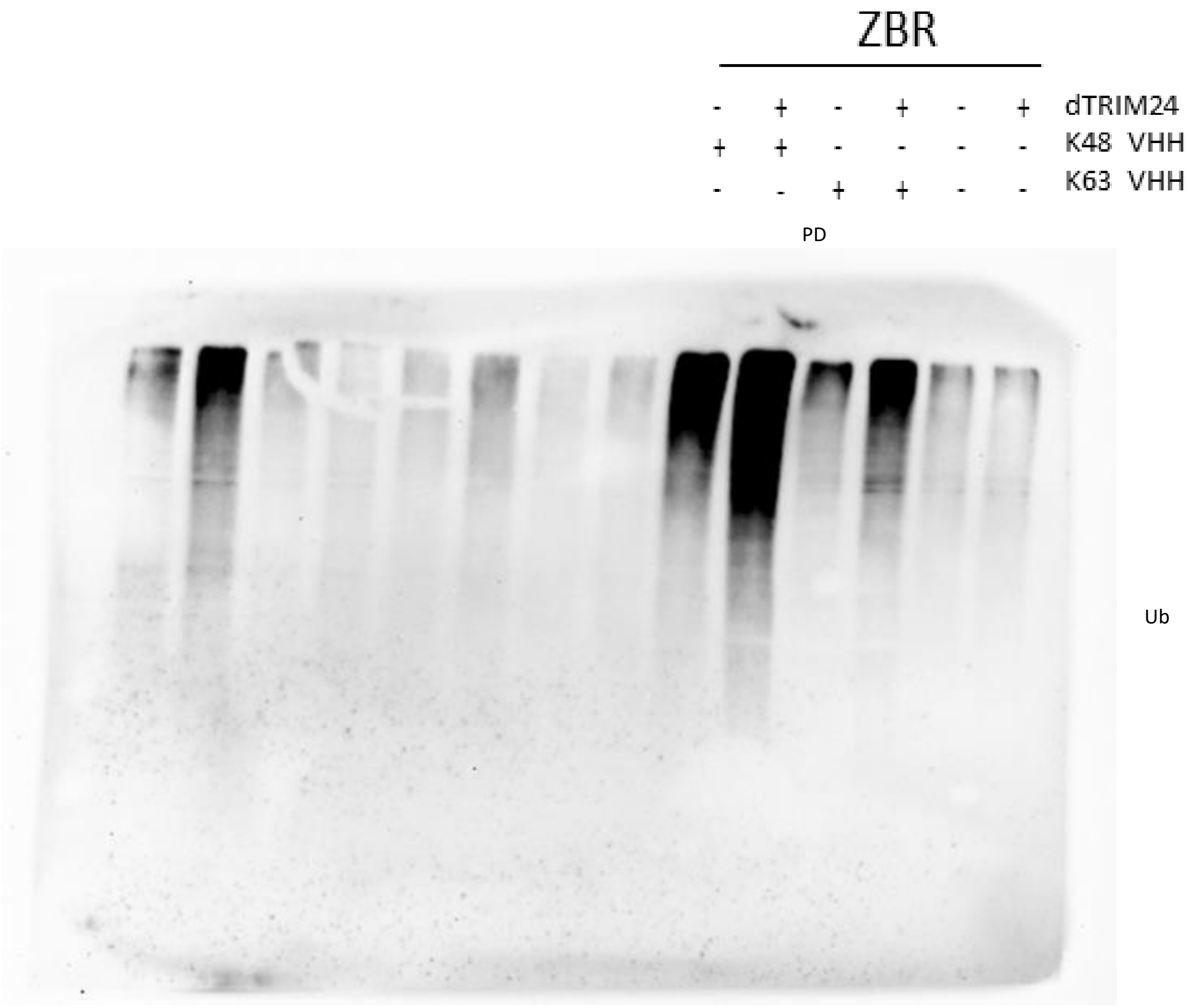
ZBR
- + - + - + dTRIM24
+ + - - - - K48 VHH
- - + + - - K63 VHH
PD
Ub

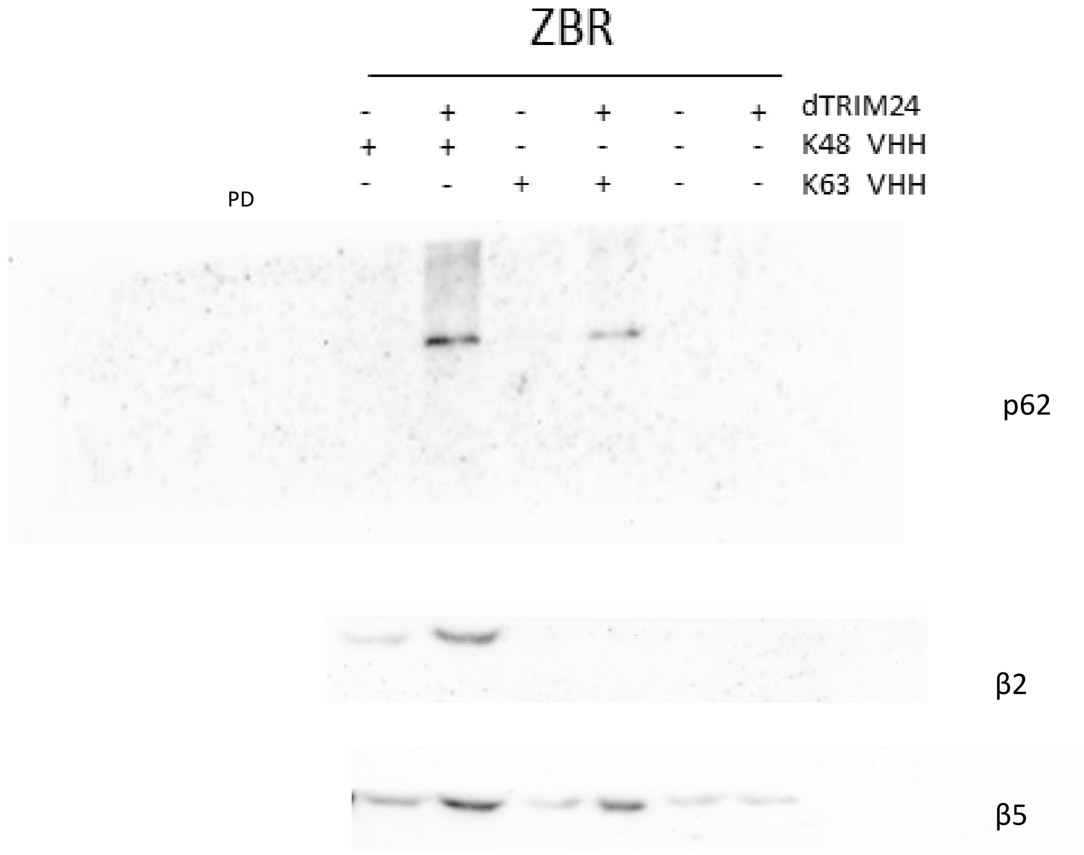
ZBR
- + - + - + dTRIM24
+ + - - - - K48 VHH
- - + + - - K63 VHH
PD
p62
β2
β5

Supplementary Figure 2:

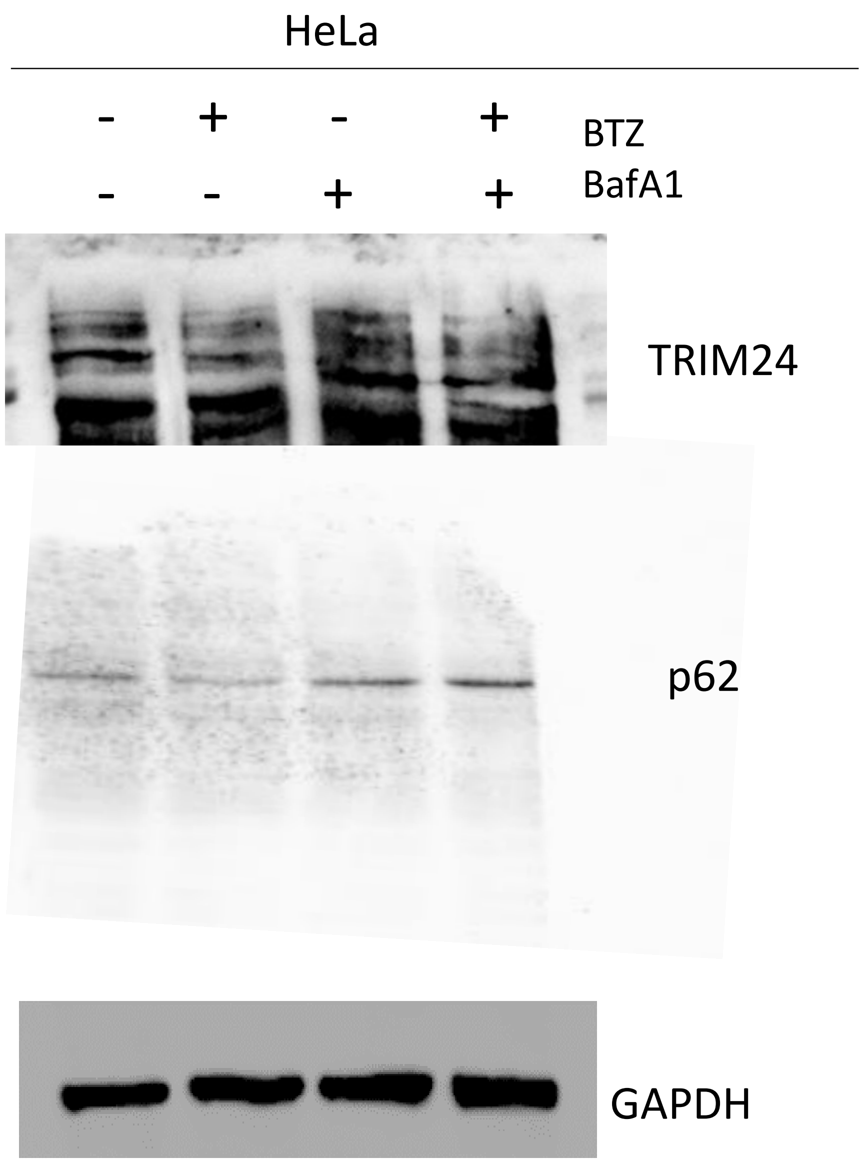

Supplementary Figure 3:

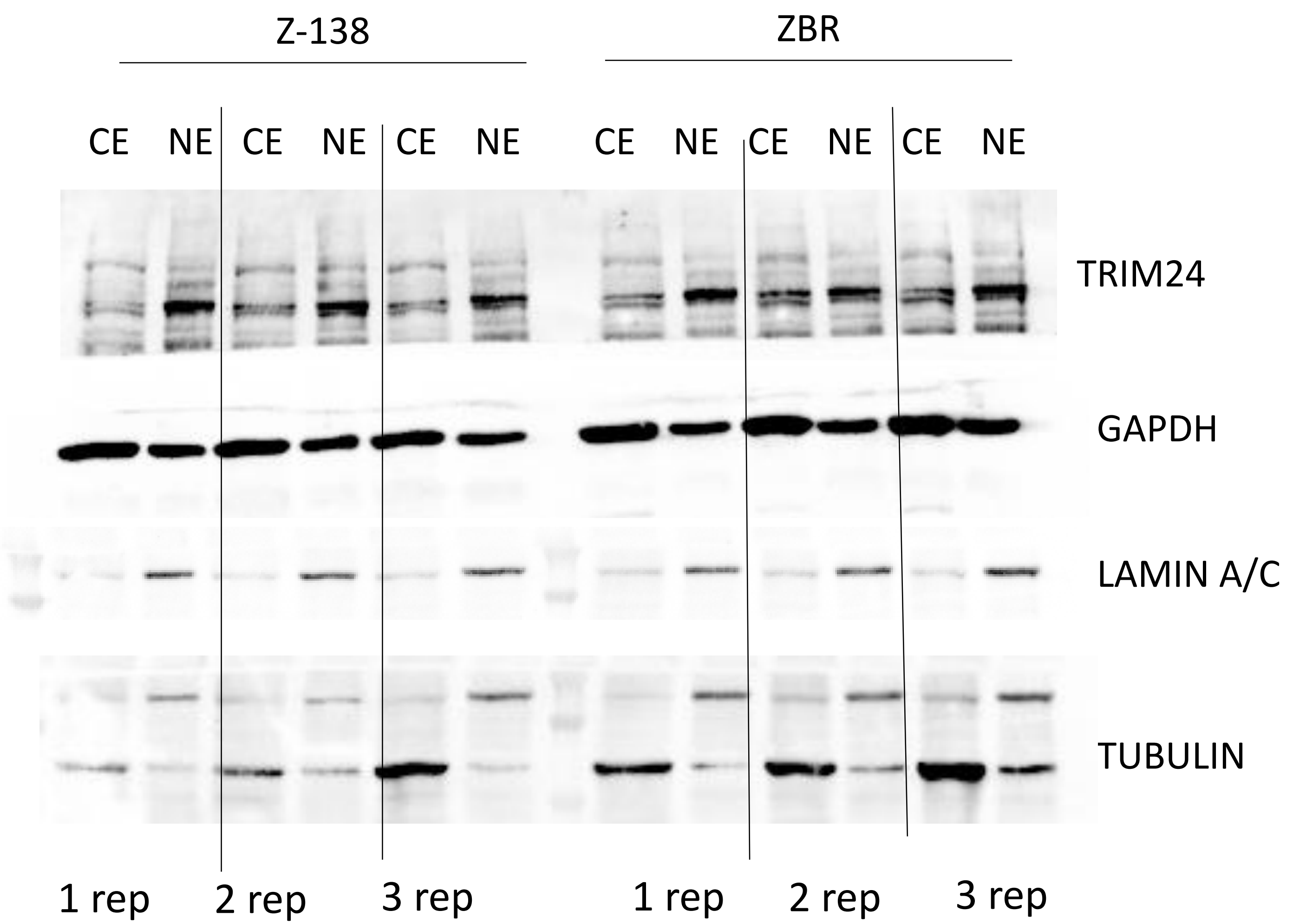

Supplementary Figure 4:

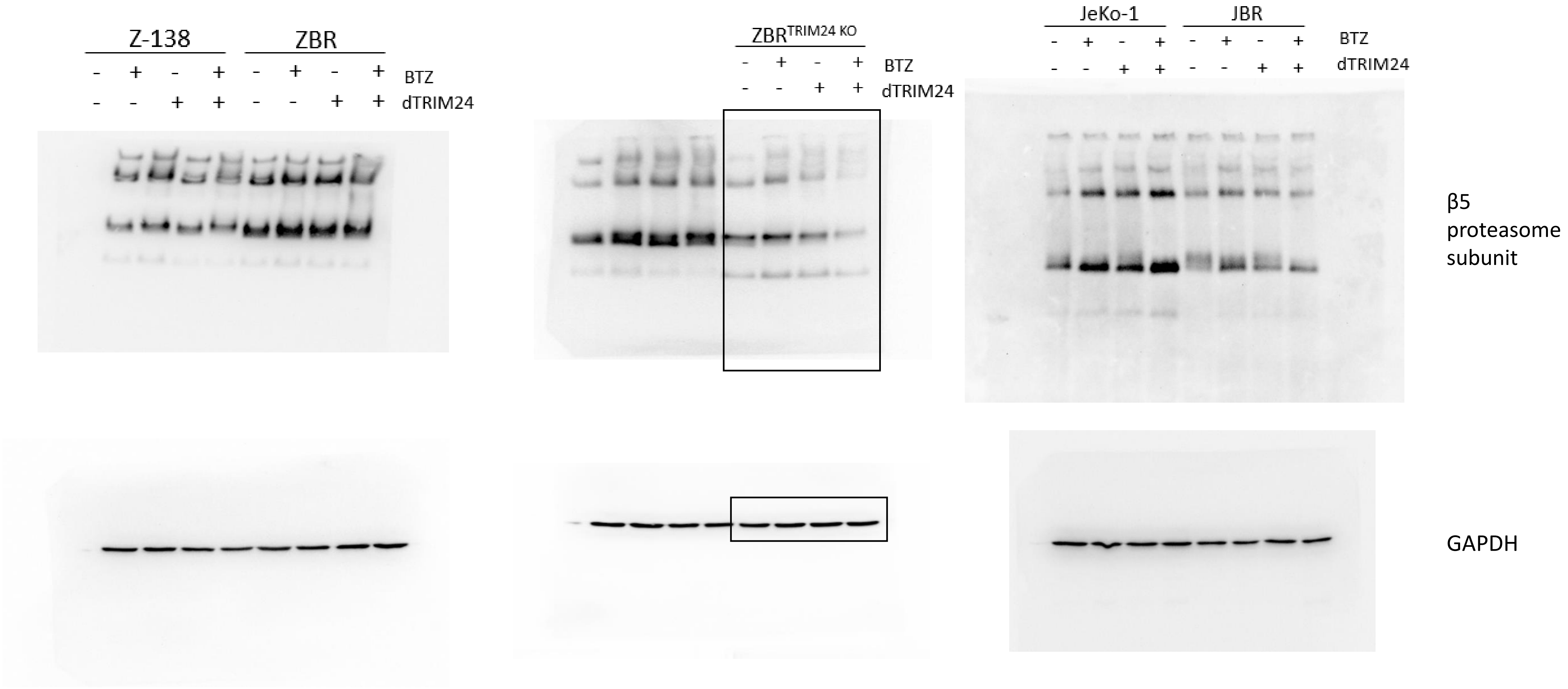

Supplementary Figure 5:

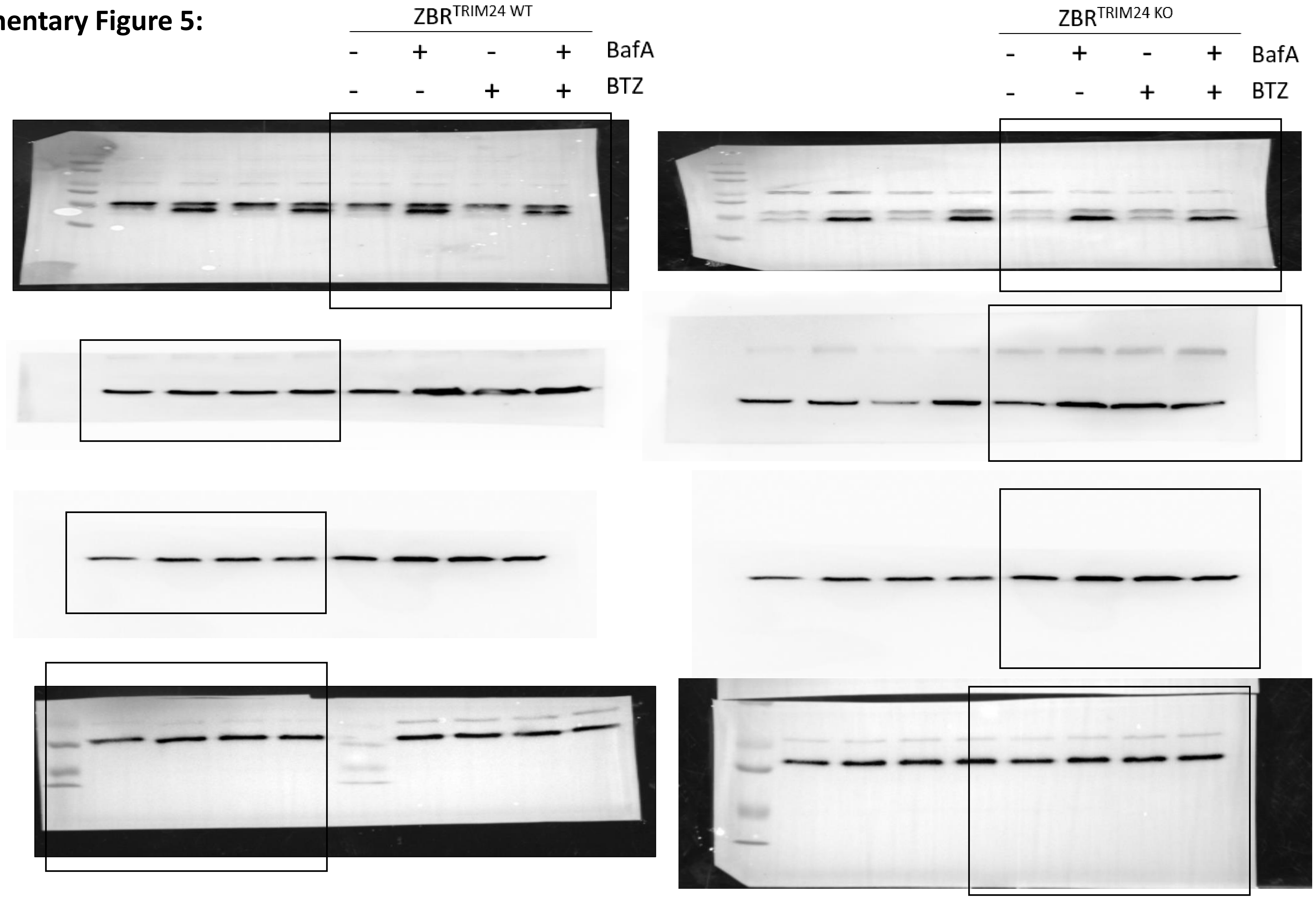

Supplementary Figure 5:

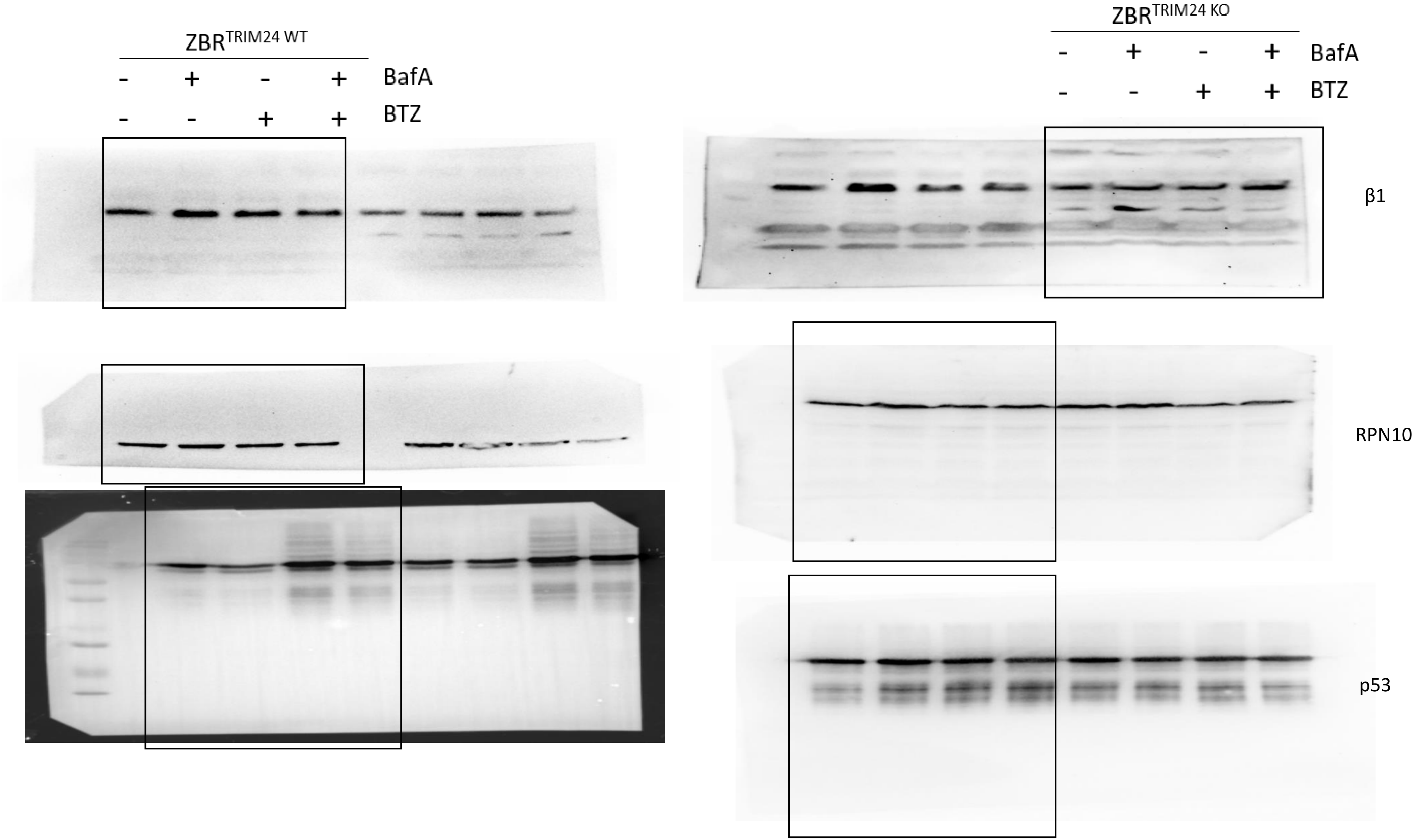

Supplementary Figure 5:

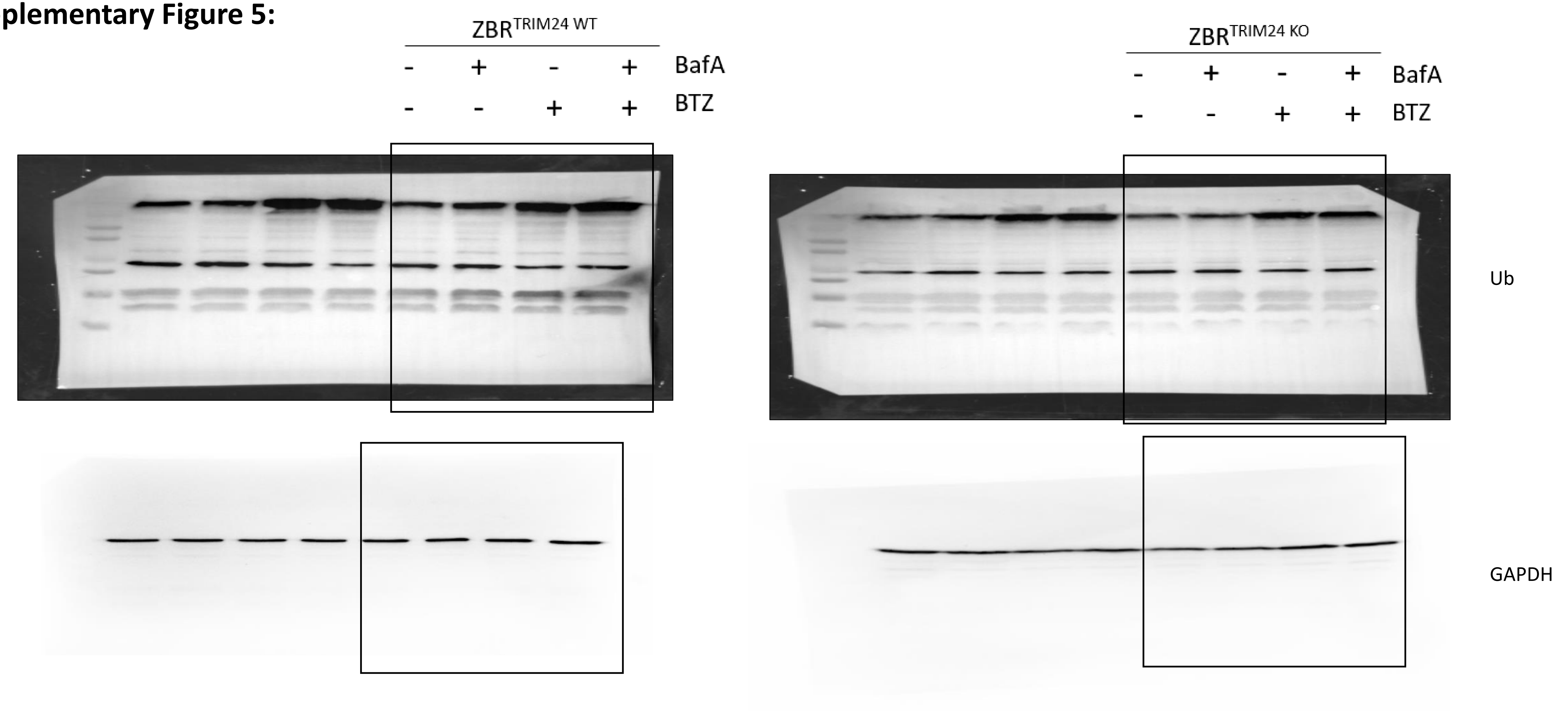

Supplementary Figure 6:

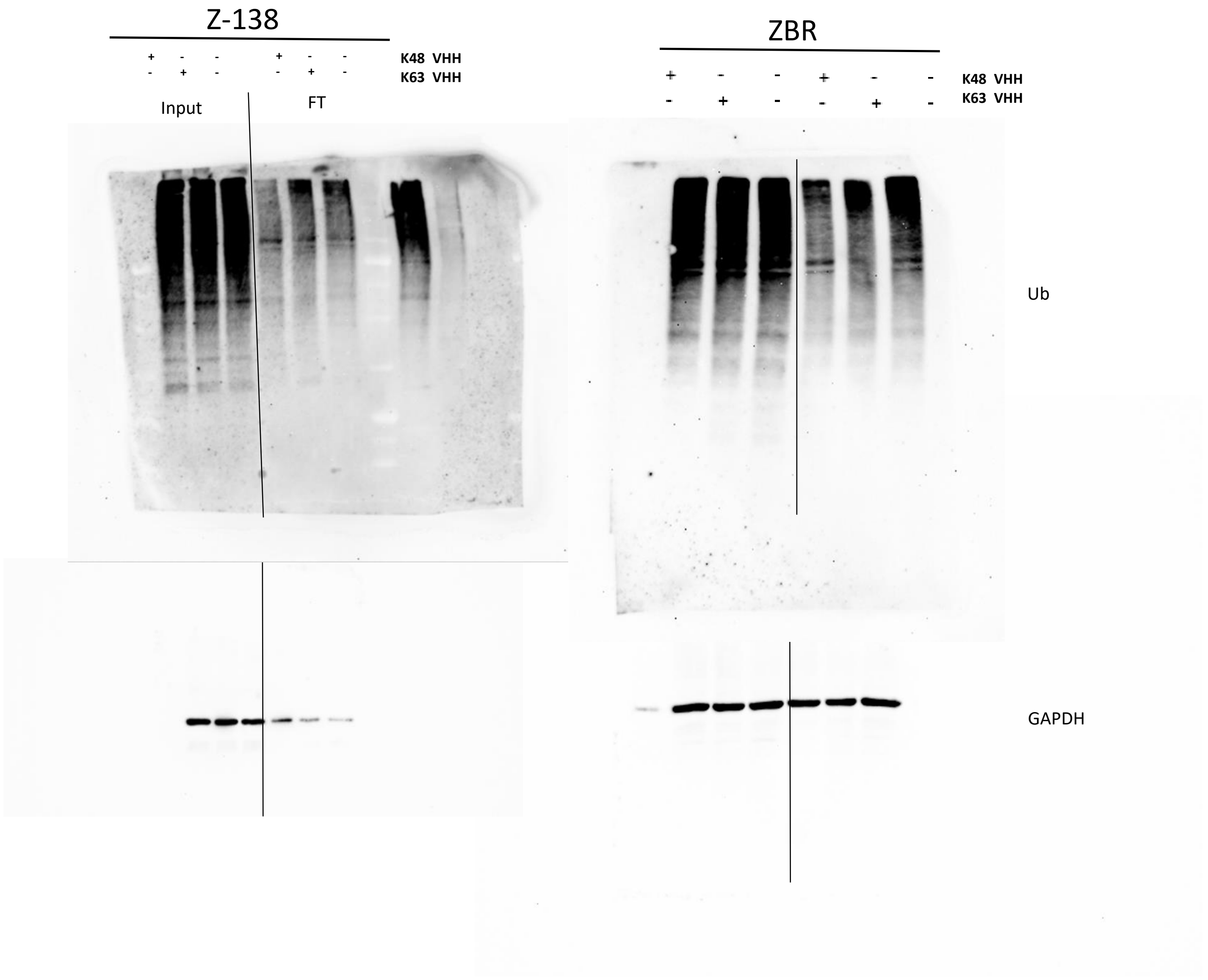

Supplementary Figure 6:

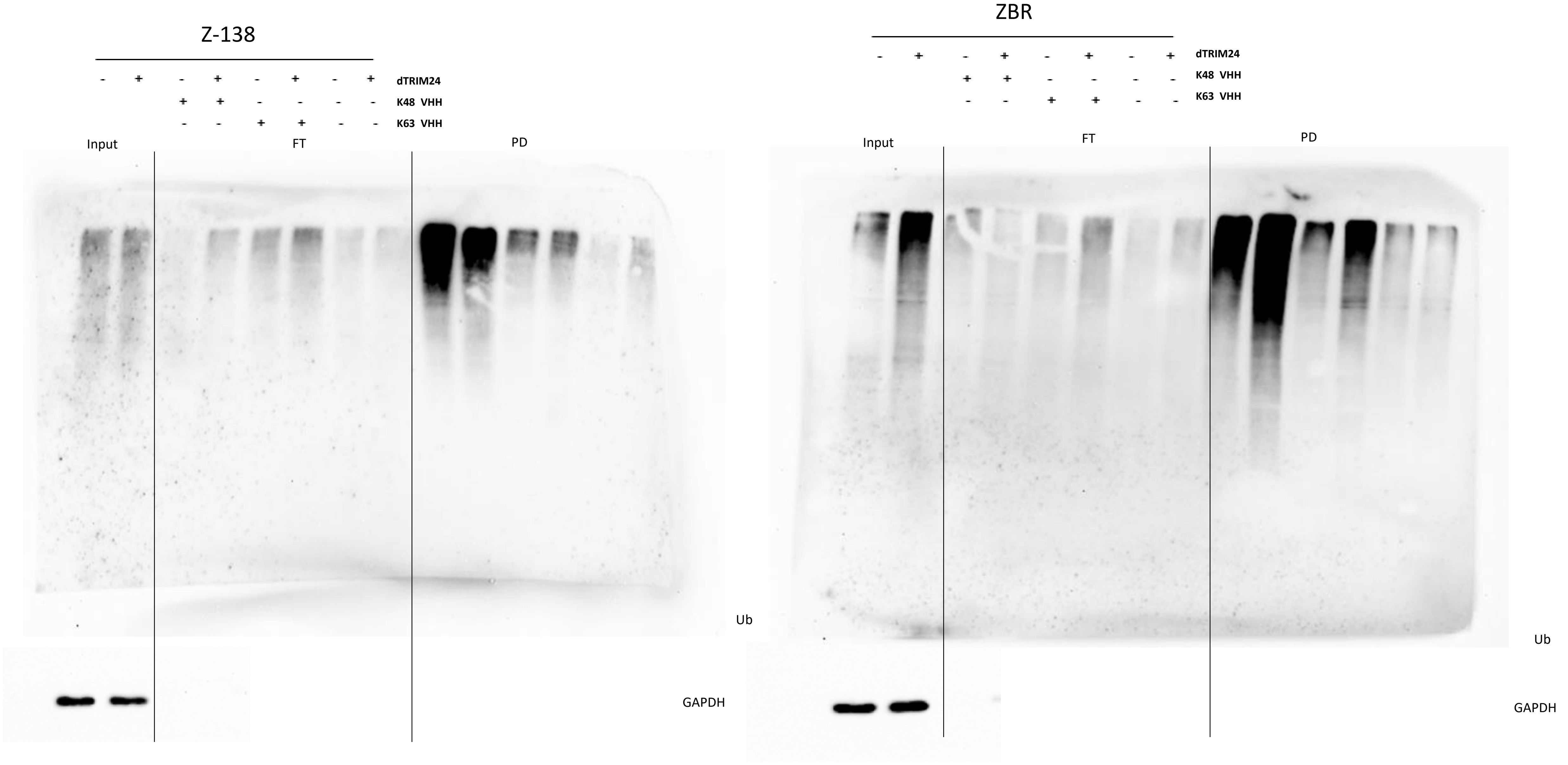

Supplement: Supplementary file 2 — Supplementary Material Original Werstern blots [file 41420_2025_2355_MOESM2_ESM.pdf]
